# Loci associated with variation in gene expression and growth in juvenile salmon are influenced by the presence of a growth hormone transgene

**DOI:** 10.1186/s12864-020-6586-0

**Published:** 2020-02-27

**Authors:** Erin Kathleen McClelland, Michelle T. T. Chan, Xiang Lin, Dionne Sakhrani, Felicia Vincelli, Jin-Hyoung Kim, Daniel D. Heath, Robert H. Devlin

**Affiliations:** 1 Fisheries and Oceans Canada, 4160 Marine Drive, West Vancouver, BC V7V 1N6 Canada; 2 EKM Consulting 730 Drake St, Nanaimo, BC V9S 2T1 Canada; 3 Great Lakes Institute for Environmental Research, University of Windsor, 401 Sunset Ave, Windsor, ON N9B 3P4 Canada; 4 Korea Polar Research Institute (KOPRI), 26, Songdomirae-ro, Yeonsu-gu, Incheon, 21990 South Korea

**Keywords:** Transgenic fish, Coho salmon, Growth hormone, Body size, Genome-wide association study, Genotyping-by-sequencing, SNPs

## Abstract

**Background:**

Growth regulation is a complex process influenced by genetic and environmental factors. We examined differences between growth hormone (GH) transgenic (T) and non-transgenic (NT) coho salmon to elucidate whether the same loci were involved in controlling body size and gene expression phenotypes, and to assess whether physiological transformations occurring from GH transgenesis were under the influence of alternative pathways. The following genomic techniques were used to explore differences between size classes within and between transgenotypes (T vs. NT): RNA-Seq/Differentially Expressed Gene (DEG) analysis, quantitative PCR (qPCR) and OpenArray analysis, Genotyping-by-Sequencing, and Genome-Wide Association Study (GWAS).

**Results:**

DEGs identified in comparisons between the large and small tails of the size distributions of T and NT salmon (NT_Large_, NT_Small_, T_Large_ and T_Small_) spanned a broad range of biological processes, indicating wide-spread influence of the transgene on gene expression. Overexpression of growth hormone led to differences in regulatory loci between transgenotypes and size classes. Expression levels were significantly greater in T fish at 16 of 31 loci and in NT fish for 10 loci. Eleven genes exhibited different mRNA levels when the interaction of size and transgenotype was considered (IGF1, IGFBP1, GH, C3–4, FAS, FAD6, GLUT1, G6PASE1, GOGAT, MID1IP1). In the GWAS, 649 unique SNPs were significantly associated with at least one study trait, with most SNPs associated with one of the following traits: C3_4, ELA1, GLK, IGF1, IGFBP1, IGFII, or LEPTIN. Only 1 phenotype-associated SNP was found in common between T and NT fish, and there were no SNPs in common between transgenotypes when size was considered.

**Conclusions:**

Multiple regulatory loci affecting gene expression were shared between fast-growing and slow-growing fish within T or NT groups, but no such regulatory loci were found to be shared between NT and T groups. These data reveal how GH overexpression affects the regulatory responses of the genome resulting in differences in growth, physiological pathways, and gene expression in T fish compared with the wild type. Understanding the complexity of regulatory gene interactions to generate phenotypes has importance in multiple fields ranging from applications in selective breeding to quantifying influences on evolutionary processes.

## Background

Domestication and artificial selection have long been used to increase size and growth rates of fishes and other vertebrates used for food production. More recently, creation of growth enhanced transgenic organisms through introductions of growth hormone (GH) gene constructs has been the subject of research in many fish species [1–4]. Growth regulation is a complex process influenced by genetic, cellular and environmental factors. In fishes, growth is mediated primarily via the growth hormone (GH)/insulin-like growth factor-I (IGF-I) pathway [5, 6], and introduction of a GH construct in some species has resulted in greater than 30-fold increases in the size-at-age of transgenic fish [1, 7, 8], with more modest gains in other species [9].

In salmon, GH plays a critical role in somatic growth through promotion of protein synthesis, feed intake, and feed-conversion efficiency [5, 10, 11]. In addition, GH and IGF-1 are involved in many other processes in salmon, including reproduction, feeding behaviours, predator avoidance, and osmoregulation [5, 12]. Effects of GH overexpression, relative to wild type, have also been found to be highly influenced by environmental conditions and by genotype by environment interactions [13, 14].

Recent studies comparing transgenic and non-transgenic salmon have examined the role of GH in regulating genes involved in growth. Genes involved in the GH/IGF-I pathway exhibit differential expression between wild-type and transgenic coho salmon (*Oncorhynchus kisutch*), with greatly increased expression of GH and IGF-I in the latter, and multiple differences between genotypes in other pathways, including transcription, amino acid metabolism, respiration, stress/immune function, lipid metabolism/transport, brain/neuron function, and carbohydrate metabolism [10, 15–17]. Levels of myostatin 2, a protein involved in muscle development and growth, was found to vary between transgenic and wild-type salmon, with higher levels in red muscle of transgenic fish and lower levels in white muscle [18]. Genes involved in appetite and feeding response (e.g., AgRP1) are also strongly differentially expressed (approximately 15-fold) in the brain and pituitary gland of transgenic coho salmon compared with wild-type fish [19].

Comparisons of the effects of GH transgenesis among strains with different genetic backgrounds has found variable growth responses. For example, a highly domesticated (fast-growing) strain of rainbow trout (*Oncorhynchus mykiss*) showed little or no increase in size compared with wild strains following introduction of the GH transgene [7], whereas in a wild-type (slow-growing) trout strain variable responses were detected [17]. In contrast, in coho salmon, additive effects of domestication and GH transgenesis were observed [16]. Similar strain effects have also been observed in GH transgenic mice [20]. Thus, the genetic background into which the GH transgene construct is introduced appears to influence observed changes in phenotype. Recent studies have indicated that phenotypic effects of GH transgenesis and domestication may arise from similar influences on gene expression and physiological pathways. Indeed, previous measurements in domesticated salmonids have found elevated levels of GH and IGF-I relative to wild type [21, 22] as occurs in GH transgenic fish [10], indicating this growth-regulating pathway is affected in similar ways by these two types of genetic change. However, it is not clear if all types of fast-growing strains, or all fast-growing individuals within strains, exhibit similar phenotypes due to parallel changes in gene expression and physiology.

In order to more directly examine if genetic background affects the phenotypic outcomes of GH transgenesis, and whether such influences affect phenotype in non-transgenic (NT) and GH transgenic (T) siblings in the same or distinct ways, we identified differentially expressed genes (DEGs) between fish size classes (large vs. small) within both T and NT salmon. The analysis examined whether the presence of a GH transgene affected expression (mRNA levels) of genes associated with growth (and other pathways of interest) in GH transgenic and non-transgenic fish. We further performed a Single Nucleotide Polymorphism (SNP)-based genome-wide association study (GWAS) to identify loci that affected body size as well as the expression of an array of genes involved in growth and other pathways affected by GH. Specifically, we examined whether the same or different regulatory loci are involved in controlling body size and gene expression variation between T and NT fish, with the objective of assessing whether the physiological transformations occurring from GH transgenesis are under the influence of alternative gene regulation pathways than those affecting size variation in NT salmon. The analysis found multiple regulatory loci affecting gene expression between fast-growing and slow-growing fish within T or NT groups, but few such regulatory loci were found to be shared between NT and T groups. These data have revealed how GH overexpression alters the regulatory responses of the genome to the shift in growth, physiological pathways, and gene expression associated with GH transgenesis.

## Results

### RNA-Seq, differentially expressed genes, and GO analysis

From the RNA-Seq analysis, an average of 14,529,510; 14,492,284; 14,298,225; and 14,099,226 RNA sequencing reads were detected in the technical replicates for NT_Large_, NT_Small_, T_Large_ and T_Small_, respectively.

**Fig. 1:**
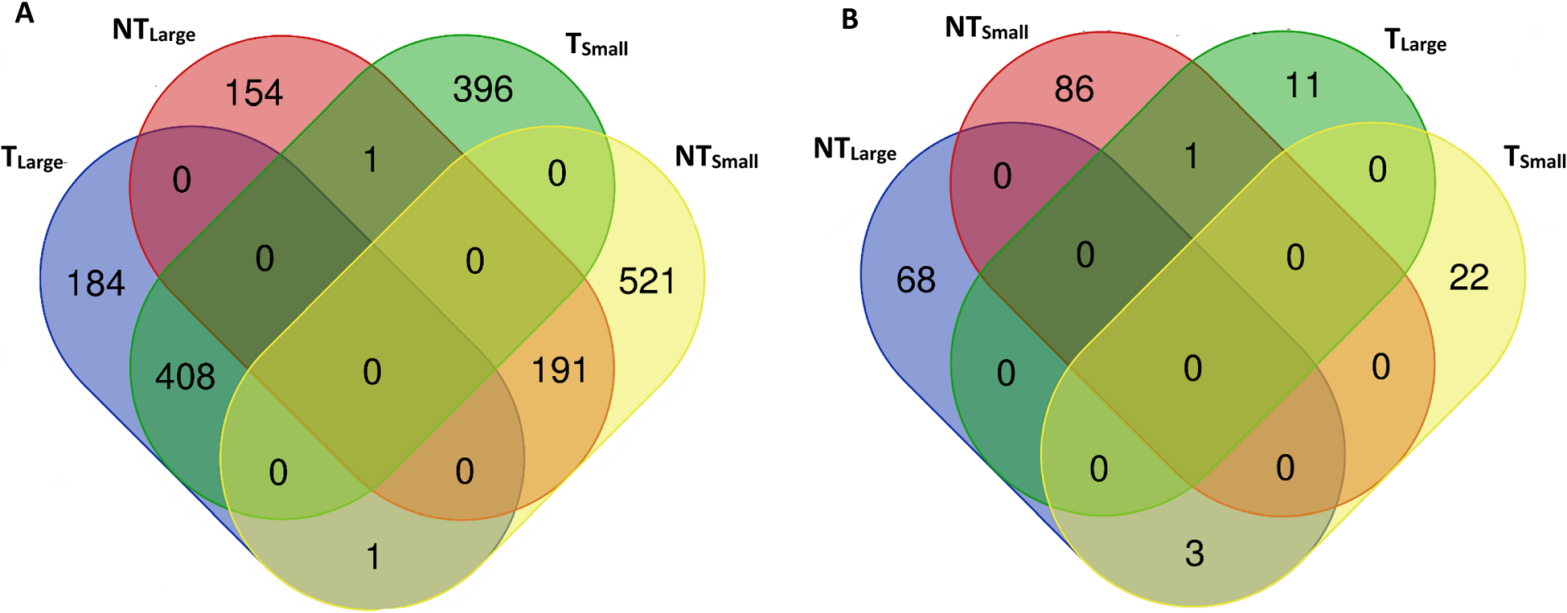
Venn diagrams showing unique and shared Differentially Expressed Genes (DEGs) between fish identified in comparisons by size or by transgenotype (large transgenic, T_Large_; small transgenic, T_Small_; large non-transgenic, NT_Large_; and small non-transgenic, NT_Small_). **a** DEGs identified in comparisons within sizes across transgenotypes (T_Small_ and NT_Small_ or T_Large_ and NT_Large_) that had higher expression in the indicated group and an indication of whether these DEGS were unique or shared with other groups. For example, 346 DEGS were overexpressed in NT_Large_ compared with T_Large_; 191 of these were also overexpressed in NT_Small_ in a comparison with T_Small_. In other words, 191 DEGS were overexpressed in non-transgenic fish as compared with size matched transgenic fish. **b** DEGs identified in comparisons within transgenotypes (NT_Small_ vs. NT_Large_ and T_Large_ vs. T_Small_) that had higher expression in the indicated group and an indication of whether these DEGS were unique or shared with other groups

DEGs from RNA-Seq analyses comparing fish from the large and small tails of the size distributions of T and NT salmon (NT_Large_, NT_Small_, T_Large_ and T_Small_) spanned a broad range of biological processes, indicating a wide-spread influence of the transgene on gene expression. However, the response to the transgene differed between size groups. In a comparison of T_Large_ and NT_Large_ fish, 939 genes were found to be differentially expressed with a greater than 3-fold change in expression (Supplemental Material Table S1); of these, 593 genes had higher expression in T_Large_ fish while 346 had higher expression in NT_Large_. In contrast, 1518 genes were differentially expressed between T_Small_ and NT_Small_ fish (Table S2); 805 DEGs had higher expression in T_Small_ and 713 had higher expression in NT_Small_. Of the 346 genes that were overexpressed in NT_Large_ fish, 191 were also overexpressed in NT_Small_ fish in the comparison with T_Small_ (Fig. 1a). Similarly, 408 genes were overexpressed in T fish (i.e., in T_Large_ when compared with NT_Large_ and in T_Small_ when compared with NT_Small_; Fig. 1a).

DEGs were identified in comparisons between size groups (Large vs. Small) within transgenotypes (T or NT), albeit considerably fewer than between transgenotypes (T vs. NT). In a comparison between T_Large_ and T_Small_, only 37 DEGs were identified, of which 12 genes were more highly expressed in T_Large_ and 25 genes were more highly expressed in T_Small_ (Table S3, Fig. 1b). A greater number of DEGs were identified in comparisons of large and small NT fish, with 87 more highly expressed in NT_Small_ and 71 more highly expressed in NT_Large_ (Table S4, Fig. 1b). No DEGs were consistently upregulated in large fish across transgenotypes or in small fish across transgenotypes (Fig. 1b).

**Fig. 2:**
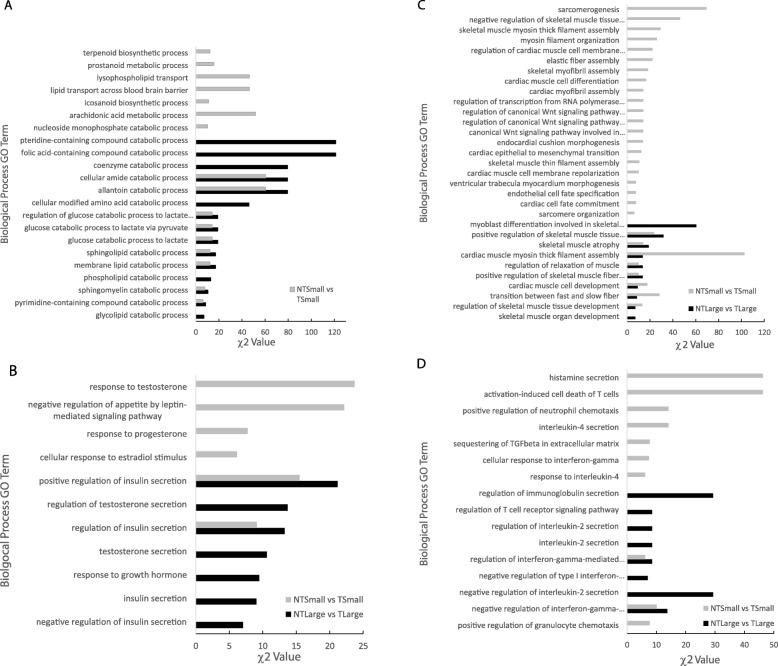
Gene Ontology (GO) Biological Process categories for the differentially expressed genes (DEGs) identified in comparisons between transgenotypes (transgenic fish, T, and non-transgenic fish, NT) for large and small fish. **a** GO terms associated with catabolism and metabolism/biosynthesis of fatty acids; **b** Endocrine control; **c** Sacromerogenesis; and **d** Immune response (see Supplemental Materials Figure S1 for complete set of DEGs)

Gene Ontogeny (GO) analysis was used to compare biological processes affected by the presence of the transgene. In the comparison between NT_Large_ and T_Large_, DEGS were assigned to 547 Biological Process GO terms (Table S5). The numbers of DEGs differed significantly from expectation for 204 GO terms (χ^2^; *p* < 0.05); for 194 terms, the number of observed DEGs was significantly greater than expected (Table S5). In the comparison between NT_Small_ and T_Small_, DEGs were assigned to 609 Biological Process GO terms (Table S5). A total of 197 categories differed significantly from expectation (χ^2^; *p* < 0.05), with 184 of these having more DEGs observed than expected. The shared biological processes in the comparison of large and small fish across the two transgenotypes reflect the genotype differences that are not due to differences in body size (Figure S1, Table S5). Processes that differed between transgenotypes included regulation of cell cycle progression and cell division, altered DNA replication, increased catabolism of essential macromolecules and changes in endocrine control (Fig. 2a, b, Figure S1).

Overexpression of growth hormone also led to changes in distinct regulatory pathways between transgenotypes at both ends of the body size spectrum. When T_Large_ fish were compared to NT_Large_ fish, we observed enrichment of genes in pathways that regulated DNA repair; DNA damage sensing mechanisms; demethylation; and responses to hyperosmotic salinity, fungus, light and UV exposure (Fig. 2, Table S5). In contrast, the comparison of T_Small_ and NT_Small_ yielded enriched GO terms associated with metabolism and biosynthesis of various fatty acids; development of skeletal muscles (sarcomerogenesis); response to immune stresses (interferon-gamma) and toxic stresses (unfolded protein, cadmium ion); regulation of macromitophagy; and sensory perception of pain (Fig. 2a, c, d, Table S5).

**Fig. 3:**
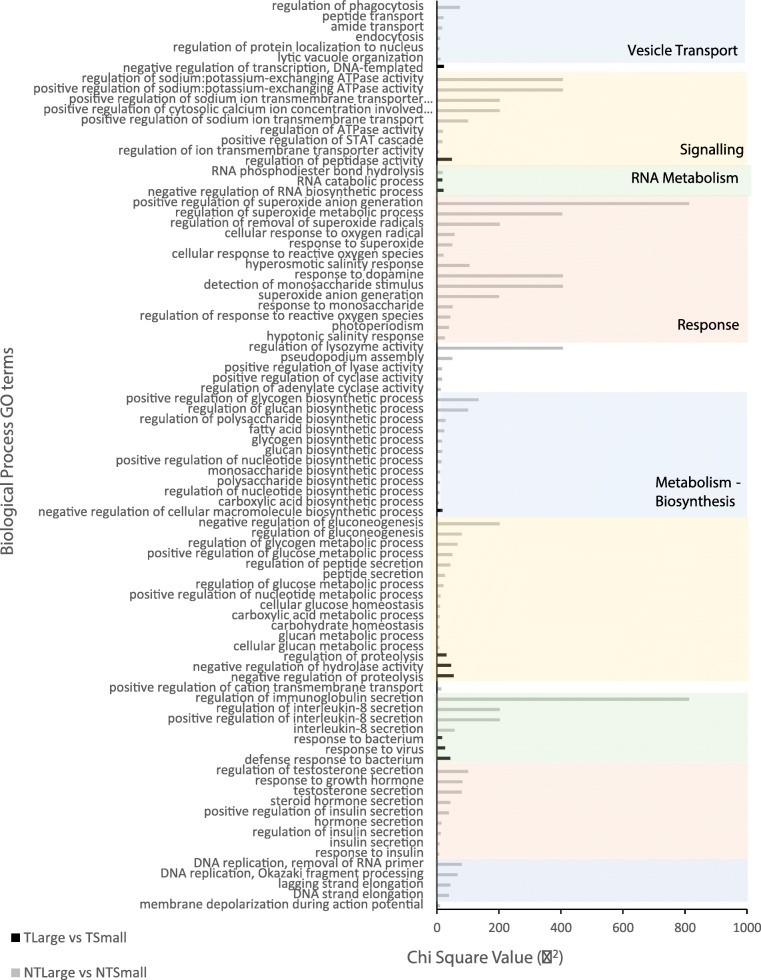
Gene Ontology (GO) Biological Process categories for the differentially expressed genes identified in comparisons between large vs. small fish within transgenotypes (transgenic fish, T, and non-transgenic fish, NT)

DEGs assigned to Biological Process GO terms were assessed for size classes within transgenotypes. DEGs were assigned to 152 and 24 Biological Process GO terms from comparisons between NT size groups and T size groups, respectively (Table S6). Differences between size classes were unique to each transgenotype (Fig. 3). In NT fish, DEG enrichment was most notable in pathways that affect carboxylic acid catabolism and biosynthesis; endocytosis (phagocytosis); generation of superoxide anion (activates glycolysis); regulation of immunoglobulin secretion; salt tolerance (related to the GH affecting carbonic anhydrase II and copper tolerance); regulation of key hormone and peptide secretions; and gluconeogenesis pathways. In the T fish, differences in GO terms between body size classes were less frequent and depended on genes that negatively regulate proteolysis; hydrolysis; transcription; and RNA and cellular macromolecular biosynthetic process (Fig. 3, Table S6).

### Quantitative PCR

Transgenotype (T vs. NT) had a strong influence on gene expression of genes assayed individually. Twenty-six of the assessed genes exhibited significant differences in mRNA expression level between T and NT individuals (Table 1; gene name abbreviations are as for Table S9). Gene expression was greater in transgenic fish for 16 of those genes, while non-transgenic fish had higher gene expression for 10 genes. GH/IGF pathway genes exhibited significant differences between T and NT, and as expected, hepatic GH was not expressed at detectable levels in NT fish. GHR, IGF-I and IGF-II mRNA levels were elevated in T vs. NT, whereas IGFBPI, IGFBP2B2 and IGFIR were downregulated; these results were consistent with the overall stimulation of growth via the GH/IGF pathway. Differences between T and NT were particularly large for three genes: LEPTIN, GLK and G6PASE1. LEPTIN, was found at levels 12.1-fold and 8.4-fold greater in small and large NT fish as compared with their small and large T counterparts, respectively. Similarly, G6PASE1 was found at levels 5.8-fold and 4.8-fold higher in NT_Small_ and NT_Large_ compared with size matched T fish. In contrast, GLK expression levels were 23.1-fold and 10.2-fold higher in T_Small_ and T_Large_ than in NT fish.

**Table 1:**
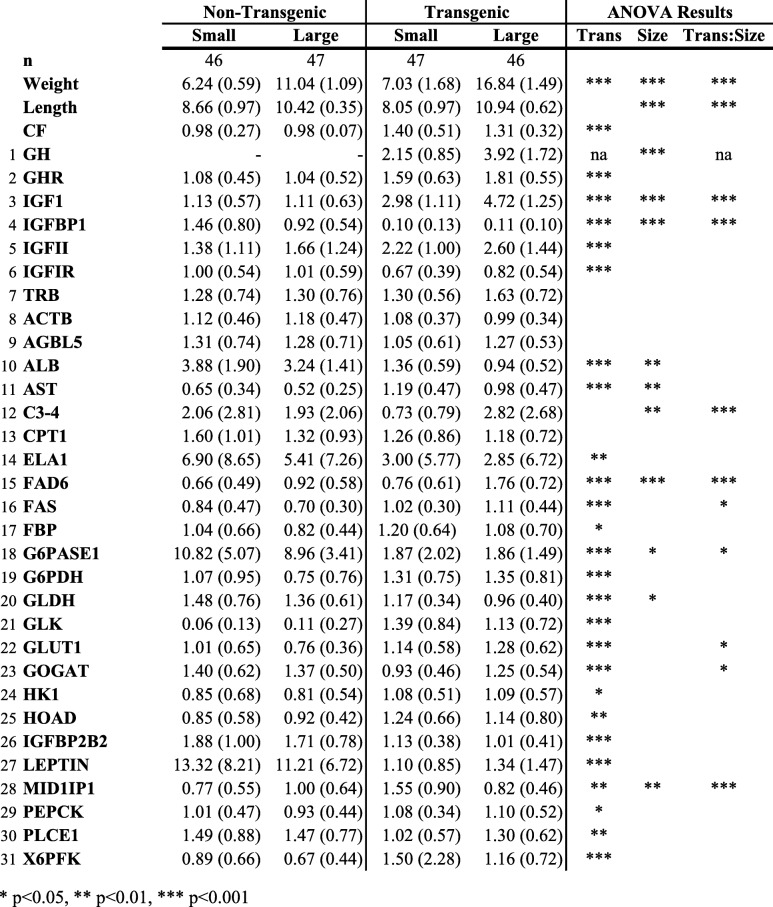
Average (standard deviation) weight (g), length (cm), condition factor (CF) and relative mRNA expression levels for genes assessed using qPCR [1–7] and Open Array [8–31] for non-transgenic and transgenic fish by size category. Results from the type II ANOVA analysis with transgenotype and size group as co-factors. Abbreviations for gene names are as for Table S9

* *p* < 0.05, ** *p* < 0.01, *** *p* < 0.001

Eleven genes exhibited different mRNA levels when the interaction of size and transgenotype was considered (IGF1, IGFBP1, GH, C3–4, FAS, FAD6, GLUT1, G6PASE1, GOGAT, MID1IP1; ANOVA *p* < 0.05; Table 1, Figure S2). There was no clear pattern for which groups differed significantly. For example, for G6PASE1 and IGFBP1 there was no difference within T fish, but T and NT fish differed and size classes differed within NT (Tukey, *p* < 0.05). In contrast, with IGF1 and C3–4 there was no difference in expression within NT fish, but T differed from NT and size classes differed within T fish (Tukey, *p* < 0.05). For FAS and GLUT1 there were significant differences between NT_Large_ and T_Large_ but not within transgenotypes or between small fish, while with GOGAT and MID1PI1, the T_Small_ group differed from all others but there were no significant differences between large fish. For ALB, AST, and GLDH, both size and transgenotype were significant factors in determining gene expression when assessed individually, although the intersection of transgenotype and size was not significant (ANOVA, *p* > 0.05).

### SNP discovery and GWAS

One transgenic fish was removed from SNP discovery due to missing sequence data. A total of 619,839 barcoded reads were considered for discovery; of these 80.2% were successfully mapped to the coho salmon reference genome. After merging multiple-aligned tags and filtering low quality reads, 62,058 unique SNPs were identified. Average read depth was 14.7x. After the additional filtering steps described above, 13,588 SNPs were considered for subsequent association analysis. SNPs were distributed fairly evenly across all 30 linkage groups with an average of 312 ± 98 SNPs per group (Figure S3). An additional 4237 SNPs were found on unassigned genome contigs and scaffolds.

For body size traits, a total of 17 SNPs were significantly associated with weight in T fish, while only 4 SNPs were associated with weight in NT fish (FDR q < 0.05; Table 2, Table S7). Similarly, 15 and 8 SNPs were associated with length in T and NT fish, respectively. In T fish, 11 SNPs were significantly associated with both weight and length, while in NT fish only 3 SNPs were significantly associated with both traits. Interestingly, condition factor was associated with 299 SNPs in NT fish but with only 7 SNPs in T fish. When fish were examined by size group (Small vs. Large), most of the significant SNPs were identified in NT_Small_ fish (Table 2). Further, 374 SNPs were associated with condition factor for NT_Small_ fish. Of these, 249 were also significantly associated with condition factor when all NT fish were examined together. A large number of SNPs were associated with length in NT_Small_ fish (311 SNPs) and there was considerable overlap in SNPs associated with CF and length (but not weight) in NT_Small_ fish (95.7% similarity).

**Table 2:**
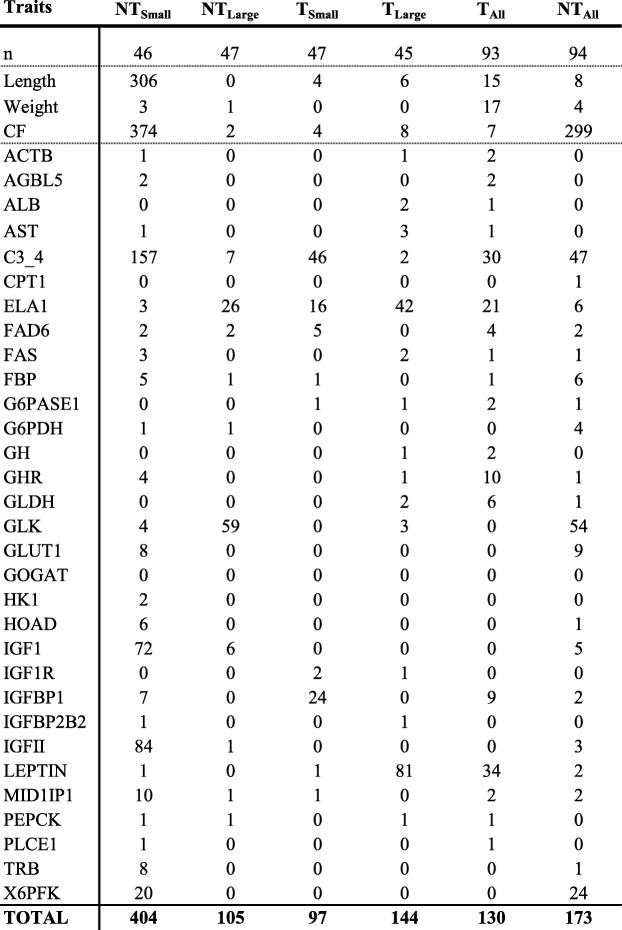
Sample size and number of SNPs associated with different traits in large and small transgenic (T) and non-transgenic (NT) fish. N indicates the sample size; trait abbreviations are as for Table S9. Note that SNPs associated with traits in transgenotype x size groups were analyzed separately from NT_All_ vs. T_All_. For details on SNP alleles and locations please see Table S7

**Fig. 4:**
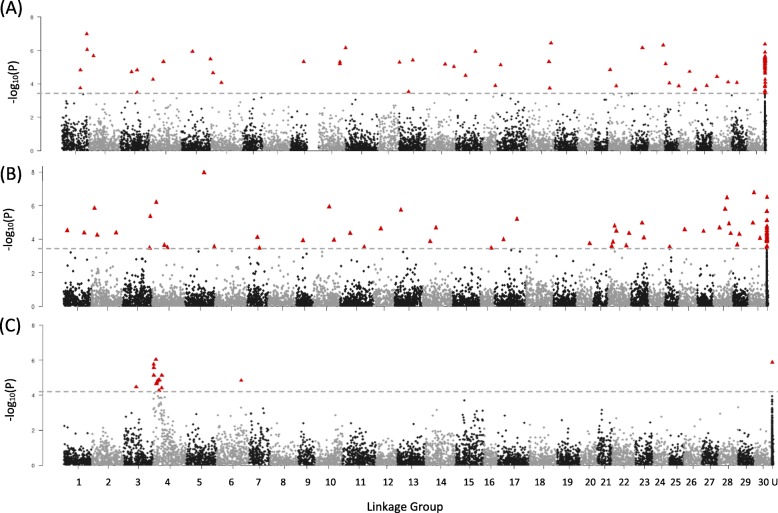
Manhattan plots of SNP number and linkage group (1–30, and unassigned (U)) for select expression traits in transgenic (T) fish. For all traits, please see Supplemental Figure S5. Significant SNPs (FDR = 0.05) are indicated by red triangles; dotted line indicates significant q value. **a** C3_4; **b** Leptin; **c** GHR (abbreviations are as for Table S1)

**Fig. 5:**
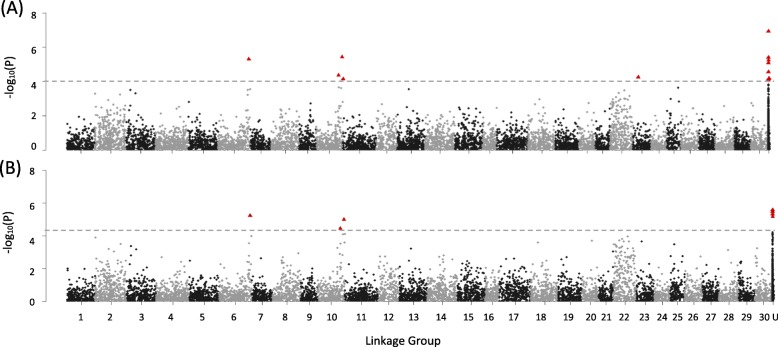
Manhattan plots of SNP number and linkage group (1–30, and unassigned (U)) for length and weight in transgenic (T) fish. For all traits, please see Supplemental Figure S6. Significant SNPs (FDR = 0.05) are indicated by red triangles; dotted line indicates significant q value. **a** Length; **b** Weight

For gene expression traits, SNPs were identified that were significantly associated with 29 of the 31 assessed genes, although SNPs were distributed unevenly across size/transgenotype groups (Table 2, Table S7). A total of 649 unique SNPs were associated with at least one trait in one of the groups, with most SNPs associated with one of the following 7 traits: C3_4, ELA1, GLK, IGF1, IGFBP1, IGFII, or LEPTIN (Table 2). Interestingly, only 2 SNPs were associated with the transgene (GH) and only in T_Large_, although more SNPs were associated with expression levels of other components of the growth hormone axis in the different transgenotype and size groups (Table 2). None of the SNPs identified as being associated with trait variation were located near (i.e., less than 1 Mb) the target gene (as determined by the current annotation in GenBank). For some traits, SNPs were broadly distributed across multiple linkage groups, while for others SNPs tended to be clustered on one linkage group (Figures S5, S6). For example, in T, SNPs for C3_4 and LEPTIN were found on 20 or more linkage groups, with 30 and 34 SNPs respectively (Fig. 4a, b), while the 10 SNPs for GHR were located primarily on linkage group 4 (Fig. 4c). For the phenotypic traits weight and length, SNPs were clustered on linkage groups 6 and 10, or on scaffold fragments (Fig. 5a, b).

**Fig. 6:**
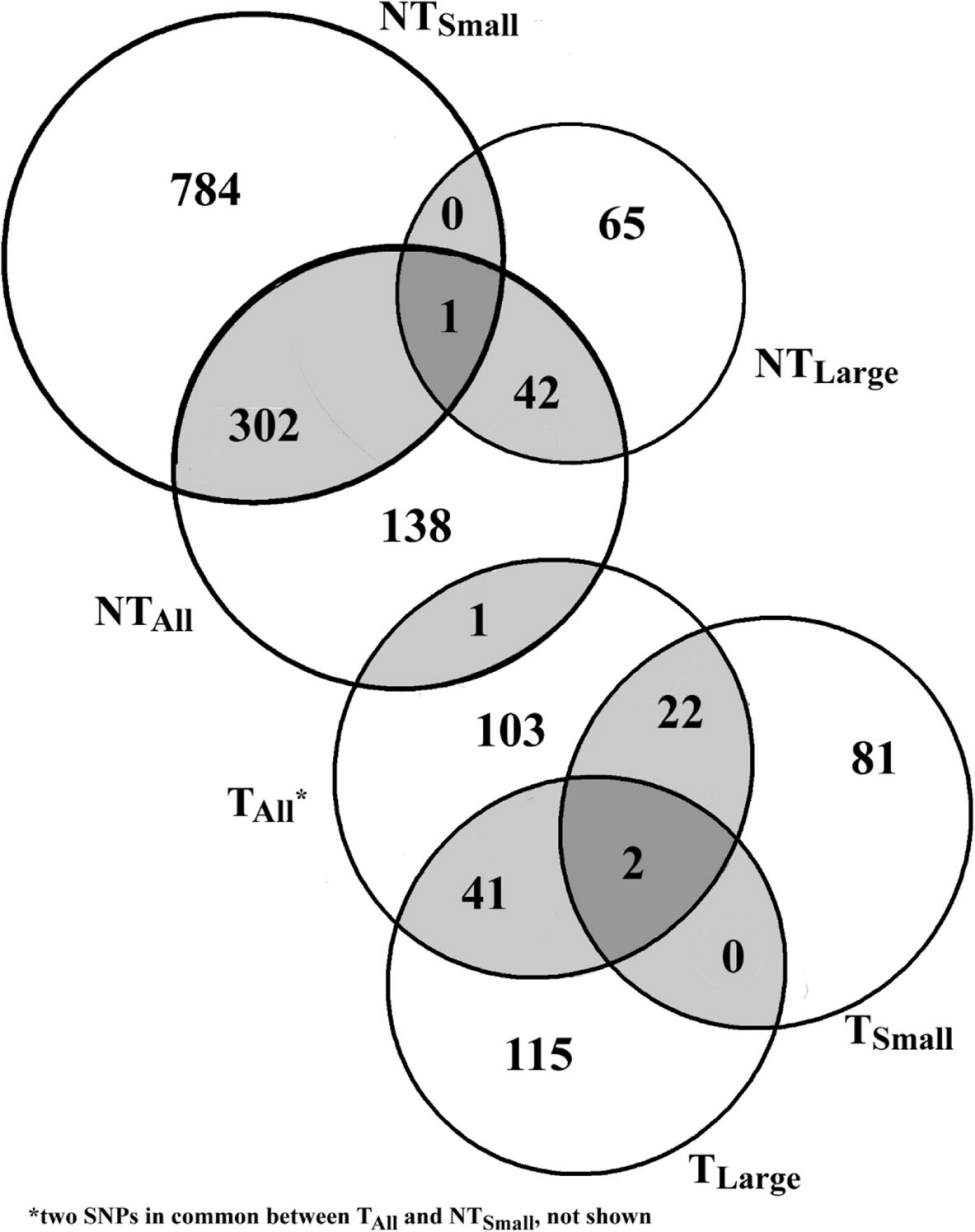
Venn Diagram of the number of significant SNPs associated with all phenotypes in each size and transgenotype category and shared between groups. T: transgenic, NT: non-transgenic

Despite both T and NT groups possessing the same genetic variation on average, only 1 SNP significantly associated with at least one phenotype was found to be in common between T and NT fish when large and small fish were analyzed together. Further, no SNPs were found in common between transgenotypes when associations were analyzed within size categories (Fig. 6). Within transgenotypes, T_Small_ and T_Large_ fish shared only 2 SNPs in common while NT size groups had only 1 SNP in common (Fig. 6, Table S9). A total of 440 SNP markers were associated with at least two traits within the same analysis, i.e., fish grouped by size or comparisons between transgenotypes only (e.g., SNP S1_495516192 was associated with both ACTB and AST in T_Large_ and also with C3–4 in NT_Small_ fish; Table S9). Of these markers, 93% were significantly associated with more than one trait in NT_Small_. The majority of the markers associated with multiple traits were explained by the correlation between CF and Length, with 280 SNPs associated with both of those traits but not with others. One SNP, S1_1372865070, located on chromosome 23, was associated with 5 traits (ALB, GLDH, GLK, IGFBP2B2, and PEPCK) in T_Large_ fish and with 4 traits (ALB, GLDH, Length and Weight) in T fish when size was not considered.

## Discussion

Here we used multiple methods (RNA-Seq/DEG analysis; quantitative PCR; GBS with SNP discovery; and GWAS) to examine the effects of a GH transgene on body size phenotypes and on expression of key growth-related genes in coho salmon. We further examined how the GH transgene influenced trans-acting regulatory loci affecting variation at these morphological and gene expression traits. It is well documented that the presence of the GH transgenesis results in overexpression of growth hormone in transgenic fish with correspondingly faster growth rates and larger average size (e.g. 10). Here too, we found that transgenic fish had elevated growth rate as well as a higher average condition factor.

### Differentially expressed genes and GO analysis

Although multiple studies have reported the physiological difference between non-transgenic and GH transgenic coho salmon (e.g., 4), it is not well understood which pathways are involved in causing size variation within and between transgenotypes (as opposed to being a secondary consequence of growth modification). Using DEG analysis, we found changes to a diversity of pathways that were unique to each transgenotype. Considerably more DEGs were identified in comparisons between transgenotypes (i.e., between NT_Small_ and T_Small_, or between NT_Large_ and T_Large_), than between size classes within transgenotypes. Shared differences in biological processes observed in the comparison of large fish between transgenotypes, and of small fish between transgenotypes, reflect the influence of the transgene rather than effects of body size. As expected, the present study found that overexpression of GH in transgenic fish led to negative regulation of cell cycle progression and cell division, altered DNA replication, increase catabolism of essential macromolecules and changes in endocrine control resulting in differentially expressed genes associated with accelerated developmental changes in neurogenesis, skeletal muscle, hemopoietic stem cell differentiation and cardiac muscle development.

Interestingly, overexpression of growth hormone also led to changes in gene expression between T vs. NT fish that differed between ends of the body size distribution. When T_Large_ fish were compared to NT_Large_ fish, there was enrichment of genes in pathways that regulate DNA repair, DNA damage sensing mechanism, demethylation, response to hyperosmotic salinity, fungus, light and UV. In contrast, the comparison of T_Small_ and NT_Small_ yielded enriched GO terms for metabolism of arachidonic acid and prostanoid, biosynthesis of terpenoid and icosanoids, development of skeletal muscles (sarcomerogenesis), response to immune stresses (interferon-gamma), and toxic stresses (unfolded protein, cadmium ion). There was also enrichment of GO terms for regulation of macromitophagy and sensory perception of pain in the comparison of T_Small_ and NT_Small_. We note that several of these pathways specifically respond to hyperosmotic stress and immune responses, effects observed between transgenic and wild-type fishes. For example, GH transgenic zebrafish had increased mortality when exposed to salinity stress, and all osmoregulatory genes were down regulated under hyperosmotic stress [23]. Presence of a GH transgene has also been found to influence the development of saltwater tolerance in coho salmon undergoing smoltification [24]. In the present study, we found that expression levels of C3_4, a component of the complement system involved in immune responses, differed between large and small fish; however, in non-transgenic fish, expression was elevated in small fish while in transgenic fish, expression was elevated in large fish. Transgenic coho salmon also have a dampened immune response in comparison with wild-type fish [25], including changes in baseline expression of immune related genes in the IGF system [26].

We also compared size classes within transgenotypes. The pathways that exhibit differential gene expression in large vs. small fish differ between transgenotypes (i.e., comparing NT_Large_ vs. NT_Small_ and T_Large_ vs. T_Small_) further suggest that different genomic mechanisms may be leading to the large (or small) size phenotype in transgenic vs. non-transgenic coho salmon. For example, in the comparison of NT fish, differential gene expressions were most notable in pathways that affect carboxylic acid catabolism and biosynthesis, endocytosis (phagocytosis), generation of superoxide anion (activates glycolysis), regulation of immunoglobulin secretion, salt tolerance (related to the GH affecting carbonic anhydrase II and copper tolerance), regulation of key hormone and peptide secretions, and gluconeogenesis pathways. In the comparison within T fish, DEGs between size groups was involved in negative regulation of proteolysis, hydrolysis, transcription, RNA and cellular macromolecular biosynthetic processes.

### Gene expression and morphological phenotypes

Within each of the T and NT progeny groups, there was also significant variation in the weight and length between large and small T fish, comparable to the variation seen between large and small NT salmon with the same median size. To investigate the basis of this variation, we examined GH expression among size and transgenotypes groups and found GH levels to be related both to transgenotype and body size within transgenotypes (GH was not expressed in the livers of non-transgenic fish as expected). Higher GH expression was seen in T_Large_ than in T_Small_ groups, suggesting variation in transgene expression may exist within the strain. Other genes in the growth hormone axis demonstrated differences in expression across transgenotypes, with GHR, IGF1, and IGFII being overexpressed in transgenic fish, while IGFBP1, IGF1R and IGFBP2B2 were underexpressed. For IGF1 and IGFBP1, there was also a significant interaction between transgenotype and size, with the highest expression levels in T_Large_ and NT_Small_ respectively. These results are generally consistent with findings from previous studies of transgenic coho salmon [10] and with other studies of GH and IGF binding proteins in fishes [6, 27]. GH-transgenic zebrafish exhibited an increase in expression of IGF1 and IGFII, and a decrease in IGF1R [28]. In GH-transgenic Nile tilapia, IGFBP1 was lower than in wild-type fish [29]. GH and other genes in the growth hormone axis are involved in several important traits beyond growth, including hypo-osmoregulatory changes during smoltification and upon initial entry to saltwater, regulation of sexual maturity, and in feeding behaviour and aggression (reviewed in 5). Thus, large differences in gene expression patterns are expected between GH transgenic and wild-type salmon.

Of the expression traits surveyed, 84% differed significantly between transgenic and non-transgenic fish, with one-third also differing between size categories. In addition to the genes in the growth axis discussed above, two other genes stand out as being highly differentially expressed between transgenotypes: LEPTIN and G6PASE1. Expression of G6PASE1 also differed significantly between size groups within and between transgenotypes, with the highest levels of expression occurring in NT_Small_ followed by NT_Large_. G6PASE1 is involved in regulation of carbohydrate metabolism and appears to not be under dietary control in salmonids [30]. Reduced expression of G6PASE1 in transgenic fish would suggests that these fish have limited ability to metabolize glucose in the liver or utilize this energy source. However, direct experimental assessment [31] suggests that transgenic fish may have elevated ability to use carbohydrates. Previous measures of G6PASE1/2 have found differing results depending on whether fish were in a fed or food-deprived state. In the present study and that of Abernathy et al. [32], fish were sampled while in a fed and growing state, and in both cases G6PASE mRNA levels were seen to be reduced in T relative to NT. In contrast, Panserat et al. [33] analyzed fish in a food deprived state and did not detect a difference in mRNA level for G6PASE. This discordance in results is intriguing and fosters speculation that the G6PASE gene expression changes observed may be strongly sensitive to experimental conditions and the nutritional status of the fish. Glucokinase was found to be elevated in the present study as well as in previous assessments that found enhanced potential for carbohydrate utilization in GH transgenic salmon [32, 33]. LEPTIN also had large changes in expression between transgenotypes, but not between size classes. Here we observed a decrease in LEPTIN levels in GH transgenic fish which is consistent with the known role of GH in suppressing leptin [34], as well as the findings of other studies of transgenic fish [29, 35, 36]. Leptin is considered a ‘pleiotropic hormone’ in fish with roles in regulating food intake and weight gain, development and maturation, and stress response and acclimation [37, 38].

### Genome-wide association study

The GWAS found that, in general, more SNPs were associated with traits within non-transgenic fish than within transgenic fish, and when fish were analyzed by size group within transgenotypes, most SNPs were detected in NT_Small_. However, there were almost no SNPs shared among groups and very few SNPs shared between traits, with the exception of SNPs shared between CF and length in NT_Small_. This is in accord with another recent study examining body size variation throughout the life history of transgenic coho salmon. In this case, none of 243 quantitative trait loci (QTL) for growth were found to be shared between GH transgenic and non-transgenic fish [39]. Surprisingly, a similar relationship was not observed between CF and weight, and in fact this overlap in SNPs was not observed in the comparison of T and NT where a large number of SNPs associated with CF were detected but only 8 SNPs were associated with length. However, we note that as fish were matched for length at sampling, the reduced variation between T and NT may have led to reduced power to detect SNPs associated with length.

In the current study, most gene expression traits were associated with fewer than 10 SNPs. The power of GWAS to identify a true association between SNP and trait depends on phenotypic variance, and thus rare variants, or variants of small effect size, are likely to be missed. However, some traits were associated with many more SNPs (including up to 157 for C3–4), although there was considerable variation in the number of SNPs associated with a given trait between size categories across transgenotypes. Surprisingly, only 2 SNPs associated with G6PASE1 variation were identified in transgenic fish, and only 1 SNP in non-transgenic salmon. When fish were analyzed between sizes, only 1 SNP was found in T_Small_ and T_Large_, and none were identified in NT_Small_ or NT_Large_. Unlike G6PASE1, LEPTIN expression was associated with a large number of SNPs in transgenic fish (34 in T_All_; and 81 in T_Large_ when fish were grouped by size), indicating many loci play a role in regulating this hormone in coho salmon.

To examine the influence of regulatory loci on gene expression and body-size phenotypes, we undertook a GWA analysis to identify SNPs associated with variation in these traits and further assessed these influences in the presence and absence of the GH transgene. For many of the traits associated with larger numbers of influential SNPs (*N* > 30), those SNPs tend to be broadly distributed across the genome. For example, SNPs associated with LEPTIN expression in transgenic fish were identified on 14 linkage groups (and several unassigned scaffolds). It is likely such a broad association between expression levels and the various regulatory elements rises from the diverse pathways in which LEPTIN plays a role. SNPs associated with C3–4, another gene associated with a diversity of functions [40, 41] and controlled by numerous regulatory elements [42], were also widely dispersed throughout the genome. In comparison, expression of GHR, which has a limited function in the growth hormone axis [43], was associated with just 10 SNPs in transgenic fish of which 8 were located on linkage group 4.

None of the SNPs identified here were located within the genes of the associated trait, and most were on different chromosomes from the gene being assessed, indicating the regulatory effects are for the most part acting in trans. Indeed, many studies employing a GWAS approach have not been able to identify causal sites despite extensive sequencing of areas around SNPs or other markers, indicating that in many cases the GWAS association is likely not a direct causal variant acting in a cis fashion at the gene being assessed [44]. We note that SNPs that are significantly associated with traits, regardless of the physical proximity of the SNP to the gene of interest, can still be valuable predictors of a phenotype [45].

## Conclusions

Here, we examined the impact of GH transgenesis in coho salmon relative to wild type, and have assessed genomic influences between large vs. small fish in NT and T genotypes. The results indicate that there are wide-spread regulatory influences acting to influence body size and gene expression traits, in addition to effects of GH transgenesis. The results reported here arise from one family, thus allowing us to sensitively examine differences caused by the presence of the GH transgene within the same genetic background, on average. Reducing genetic background effects is particularly useful for reducing the number of differentially expressed genes or SNPs identified using RNA-Seq approaches [46]; by decreasing genetic heterogeneity, we increase the power to detect correlations between phenotypes and specific genetic variants [45]. Thus, while the current data are family specific, the breadth of analysis supports the hypothesis that specific genetic loci influence body size and gene expression phenotypes, and that different loci are acting in transgenic salmon than act in wild type.

Trans-acting regulatory loci and/or epistatic or pleiotropic interactions are expected given that GH transgenes affect expression of proteins in complex physiological and cellular pathways, and interact with other loci and their pathways to modulate phenotypic effects. Studies with model organisms found many genetic modifiers that were capable of altering in trans the penetrance and expressivity of other loci. Indeed, evidence for trans-acting loci that act inversely to their dose to affect gene expression have been found to be wide-spread in Drosophila and plants [47–49]. In transgenic mammals, modifier loci affecting physiological processes and oncogenesis have been mapped. For example, a study in mice mapping modifier loci affecting the consequences of a mammary tumour-inducing transgene under different feeding conditions found at least 13 QTL affecting the onset, severity and metastasis, as well as QTL by diet interactions [50]. Similarly, Saito and Suzuki [51] found three modifiers affected tumour induction caused by a transgene expressing the K-rasG12V oncogene. In rats, Kantachuvesiri et al. [52] found that the effects of a transgene causing malignant hypertension were highly dependent on strain genetic background. In plants, modifiers affecting insect resistance were found to act additively with a *Bacillus thuringiensis* (Bt transgene to reduce impacts of corn earworm on soybean [53]. Thus, regulatory loci play significant roles in causing variation in traits in a variety of organisms, including those whose phenotype has been modified by transgenesis. Genetic modifiers of transgenic phenotypes likely act through interacting gene expression pathways, and via influences on the pathways their expressed proteins modulate. The precise mechanism of how regulatory effects act, and how GH transgenesis can modify these effects, is not known. We know that overexpression of GH strongly affects many gene expression and physiological pathway phenotypes, in some cases to the point of saturation [54]. As such, the influences of regulatory loci (e.g., transcription factors, proteins acting in complexes to cause epistatic effects, etc.) may be expected to have different capacities to affect pathways between GH transgenic and wild-type strains.

Transgenes are not native members of a genome that have evolved within that genomic environment. In some cases, transgenes have been found to be subject to silencing via epigenetic processes [55]. In addition, modifier loci that affect the expression of variegating transgenes have been identified in Drosophila where methylation of DNA does not occur [56]. We do know that some transgenes (e.g., GFP-expressing) in salmonids can show strong variegation that differs in extent among strains (unpublished), and we note that some transgenic individuals in the present strain (M77) do not show full growth stimulation suggesting some gene silencing mechanisms may be operating [54]. However, formally, we do not know whether the GH transgene in coho salmon used in the current study is subject to variegation affecting its expression and effects on growth, although we note that its chromosomal position has been determined to be centromeric [57] and its molecular environment is highly enriched in repetitive DNA [58], which is known to cause varied effects on gene expression. Thus, it is possible that some of the regulatory loci identified in the present study are acting to influence transgene silencing mechanisms and thus cause suppression of growth stimulation in some individuals. Further study to examine the mechanisms of transgene silencing, how this may influence interactions with other loci, and how or if these affects vary across a population, would be valuable.

The present data extend our understanding of background genetic effects beyond strain-level studies conducted previously. The findings are valuable to understand the role of background genetics in controlling phenotype specifically in GH transgenic organisms, but are also generally informative where analysis of pleiotropic effects and variable expressivity of traits are being examined. Understanding the complexity of regulatory gene interactions to generate phenotype has importance in multiple fields ranging from selective breeding to quantifying influences on evolutionary processes. The significant trans regulation of traits observed, and the finding that different loci affect phenotypic variability in GH transgenic and non-transgenic individuals, could allow selection to result in retention of different regulatory alleles between NT and T transgenotypes. Further understanding the degree to which regulatory controls differ between T and NT individuals is important for ecological risk assessments examining the potential consequences of transgenic organisms in nature where selection of variation in T organisms may not be optimal for maximum fitness of NT individuals [4, 59].

## Methods

### Experimental design and sample collection

Animals used in this study were from an outcrossed coho salmon family generated on February 28, 2012 by crossing a transgenic (T) female hemizygous for a growth hormone gene construct (strain M77) [13] and a non-transgenic (NT) male that was derived from a hatchery-supported natural population from the Chehalis River in British Columbia. This cross produced approximately a 1:1 ratio of the transgenotypes (T vs. NT) as expected for Mendelian segregation of the M77 transgene at a single insertion locus in the coho salmon genome. A total of 427 fish from this cross were reared in 200 L tanks in aerated fresh well water (10 ± 0.5 °C) under a simulated natural photoperiod, with fish densities kept below 5 kg/m^3^. Fish were fed to satiation 3 times daily with commercial salmon feed (Skretting Canada Ltd., Vancouver BC, Canada). Fish were reared at Fisheries and Oceans Canada’s West Vancouver Laboratory which is specially designed to prevent the escape of transgenic fish into the environment.

Sampling protocols have been described elsewhere [60]. In brief, fast- and slow-growing morphs were grown under the same rearing conditions, with both groups fed to satiety thrice daily using commercial salmon diets. The fish were reared together until August 14–16, 2012 when the fast-growing T fish reached a mean fork length of 9.7 cm, at which time they were sampled. NT siblings continued to be reared until November 13–15, 2012 when their mean length (9.5 cm) was approximately equal to that of the T salmon (Table S8, Figure S4). For tissue collection, coho salmon were anaesthetized using a procedure approved for use in salmon by the Canadian Council on Animal Care DFO Pacific Region Animal Care Committee (Management Procedure 3.7). Fish were netted from their rearing tank into an aerated bucket containing 200 mg/L tricaine methane sulphonate (MS222, Syndel Laboratories, Nanaimo, BC, Canada) buffered with a 2-fold weight of sodium bicarbonate in fresh well water (10 °C) to initiate aqueous absorption of the anaesthetic. Progress of anaesthetization was monitored by observing the fish’s ability to maintain equilibrium, and by noting opercular (gill cover) movement. Fish were removed from the anaesthetic bath immediately upon cessation of ventilation, at which point the unconscious animals were decapitated and tissues recovered by dissection. Lengths and weights were measured, fin clips were collected and stored in 95% ethanol for subsequent use, and liver samples were taken from the same fish and stored in RNAlater (Qiagen, Germany) at − 20 °C. Sex was determined by internal gonadal morphology and by use of a tightly linked Y-chromosomal marker (growth hormone pseudogene, GHΨ) that is genetically inseparable from the sex-determining locus [61]. Transgenotype was confirmed using a transgene-specific quantitative PCR (qPCR) test [62].

We employed an extreme phenotype sampling strategy to select fish for further genetic analysis; previous studies suggest that sampling from the tails of a phenotypic distribution has similar or increased power to detect genotype/trait associations as sampling from the full population which allows us to avoid performing costly genomic analysis on a prohibitively large number of individuals [63–65]. The roughly 20% (numbers were adjusted slightly to accommodate a 96-well format) largest and smallest fish by weight from each experimental group were selected in descending and ascending rank order, respectively, for subsequent analyses: NT_Large_ (*n* = 47; weight range 9.8–15.2 g), NT_Small_ (*n* = 46; 4.5–7.0 g), T_Large_ (*n* = 46; 15.0–22.0 g), and T_Small_ (*n* = 47; 3.0–9.3 g). We note that this strain of GH transgenic coho salmon does show a skewed distribution of size (Figure S4) such that some individuals show only partial growth enhancement, potentially arising from gene silencing mechanisms (e.g., methylation or other epigenetic influences) [54]. We included these individuals in our analyses as we wished to capture all sources of genetic influences on phenotype, but acknowledge that this added variance may have reduced our power to detect some weaker regulatory loci. However, we note that an unbalanced sampling design has been shown to be as effective as a balanced design when sampling extreme phenotypes for association mapping in at least one other study [63].

### Differential gene expression and gene ontology analysis

Differentially expressed genes (DEGs) in T and NT coho salmon were analyzed by RNA-Seq to assess the number and type of genes that were commonly affected by body size in NT and T groups. Total RNA was extracted from liver tissue using an RNeasy mini kit (Qiagen, Valencia, CA, USA) following the manufacturer’s recommendations. RNA concentration and purity were measured using a Nanodrop (Thermo Scientific, DE, USA). RNA quality was assessed with an Agilent 2100 Bioanalyzer (Agilent Technologies, CA, USA) with RIN values > 9.0.

RNA from the 12 largest and 12 smallest fish from each transgenotype were pooled for a total of 4 groups (NT_Large_, NT_Small_, T_Large_ and T_Small_). These samples were replicated for each group, and were sent for RNA sequencing and bioinformatic analysis at BGI Genomics (Cambridge, MA, USA). Polyadenylated mRNA was enriched with oligo (dT) magnetic beads, fragmented to 200 bp lengths and reverse transcribed with first-strand cDNA synthesis. The double-stranded cDNA library was then size-selected, amplified by PCR, and 2x50bp paired-end sequencing was performed using the Illumina HiSeq2000 platform (Illumina, CA, USA). Raw reads were filtered to remove adaptor sequences, ambiguous reads (reads with > 10% of bases given as N), and low-quality reads (> 50% of the read had base quality values < 5). Retained reads were then mapped to the reference transcriptome and genome of coho salmon (Assembly GCF_002021735.1; 62) with Soap2.21 [66] allowing for 2 mismatches and 3 mismatches, respectively. RNAseq data has been submitted to the NCBI database (accession: SUB6704126, PRJNA597081). Gene expression levels were calculated using the RPKM method [67]: RPKM = 10^9^ C/NL, where C is the total number of reads mapped onto a gene, N is the total number of mapped reads, and L is the sum of genes in base pairs. DEGs were then screened between technical replicates from the same group using a Poisson distribution model and between different treatment groups using the NOISeq method [68]. Groups were compared to each other by calculating the log2 ratio of normalized expression levels as follows: NT_Large_ vs. T_Large_, NT_Small_ vs. T_Small_, NT_Large_ vs. NT_Small_, and T_Large_ vs. T_Small_.

An enrichment analysis was performed to determine which Gene Ontology (GO) terms were over- or under-represented in DEG sets for groups NT_Large_, NT_Small_, T_Large_ and T_Small_. GO annotations were comprised of functional annotations from the published coho salmon transcriptome (GenBank Accession GDQG00000000.1) and BLAST searches performed against the NCBI non-redundant protein database (as of Oct. 23, 2016), as well as the SwissProt, InterProScan, and EggNOG databases, using Blast2Go v. 4.0.7 [69]. While GO-terms were not assigned to all DEGs due to the limitations of the available databases, a total of 31,151 GO annotations (mean GO-level 7.12) were considered for distribution analysis. GO distribution analysis was performed separately for each ontology (i.e., Biological Process, Molecular Function, and Cellular Component) using 6th level GO terms for DEGs with a ≥ 3-fold change in expression. The Chi-Square test was used to identify significant ontologies (*p* < 0.05) in comparison groups as follows: (χ^2^ = ∑(O − E)^2^/E), where the observed frequency (O) is the sample frequency for a particular GO term, and the expected frequency (E) is the frequency of each GO term in the transcriptome multiplied by the total number of differentially expressed genes with a greater than 3-fold change. Of the significant ontologies, those with > 1 observation were explored further. Only results for the Biological Process Category are provided here as this category is a reflection of the cellular component and molecular function categories.

The R package *gplots* [70] was used to identify the number of DEGs that were unique to each DEG set, as well as those shared among groups; results were visualized using Venn diagrams generated using an online tool available at http://bioinformatics.psb.ugent.be/webtools/Venn/. Comparisons were made between large and small fish within a transgenotype (e.g., T_Large_ vs. T_Small_) and between transgenotypes (e.g., T_Large_ vs. NT_Large_).

### Quantitative PCR

For each of the 186 fish selected for analysis (47 NT_Large_; 46 NT_Small_; 46 T_Large_; and 47 T_Small_), total RNA was extracted from liver tissue as described above; samples with an RNA integrity number (RIN) ≥7.0 were selected for quantitative PCR (qPCR). First-strand cDNA was synthesized from total RNA (0.5 μg) using the High-Capacity cDNA Reverse Transcription Kit with RNase Inhibitor (Applied Biosystems, California USA). qPCR reactions were performed with TaqMan® Fast Advance Master Mix (Applied Biosystems) on a 7500 Fast Real-Time PCR System (Applied Biosystems) under Fast conditions. A reference gene, ubiquitin, was used to normalize mRNA levels. Ubiquitin exhibited stable mRNA expression levels among experimental groups (combined sd = 0.90). Relative mRNA expression levels were calculated using the 2^-△△Ct^ method [71]. Eight genes were analyzed in individual assays: growth hormone (GH), growth hormone receptor (GHR), insulin-like growth factor 1 (IGF1), insulin-like growth factor 1 receptor (IGF1R), insulin-like growth factor II (IGFII), insulin-like growth factor binding protein 1(IGFBP1), T-cell receptor beta (TRB) and ubiquitin (UBIQ; used as a house-keeping gene).

Gene expression was also assessed for an additional 24 genes that were selected based on known or inferred function or their previously observed responses to GH treatment. TaqMan® probes and primers were designed using Primer Express Software® v3.0.1 (Thermo Fisher Scientific, MA, USA) and had a short amplicon length (50-100 bp; Table S9). The primer/probe combinations were used to print TaqMan® OpenArray® chips (Applied Biosystems, Burlington, ON, Canada) for use on a QuantStudio 12 K Flex Real-Time PCR System (Thermo Fisher Scientific, MA, USA). Each chip contained 48 subarrays of 56 through-holes, resulting in a total of 2688 through-holes per chip. Thus for each chip, 48 cDNA samples were run in duplicate for each of 24 genes on each chip. A solution comprised of cDNA (30 ng per sample), 2.5 μL of TaqMan® OpenArray® Real-Time PCR Master Mix (Applied Biosystems, Burlington, ON, Canada), and ddH2O to a total volume of 5 μL was prepared, distributed across a 384-well plate and then loaded onto the TaqMan® OpenArray® chips using the OpenArray® AccuFill System to reduce inter-assay variation. The through-holes on the chips were pre-loaded with the primer and probe sequences for each of the 24 original genes by the manufacturer. A total of 5 chips were used for 186 cDNA samples; one chip was used to run duplicate samples to determine whether there were any chip effects. ExpressionSuite Software (Thermo Fisher Scientific, MA, USA) was used to calculate raw critical threshold (CT) values. The transcription data was normalized to the average expression of the non-transgenic samples.

Significant differences in mRNA levels among NT_Large_, NT_Small_, T_Large_ and T_Small_ were determined by type II ANOVA, with transgenotype, size group and sex as factors, using the *car* package in R v. 3.1.1, with a Tukey HSD posthoc test [72, 73]. Outliers were removed prior to other analyses using the generalized extreme studentized deviate (GESD) test for outliers [74] with a = 0.01; GESD analyses were implement in R v 3.1.1.

### GBS sequencing and SNP discovery

Genotype by Sequencing (GBS) and Single Nucleotide Polymorphism (SNP) discovery were undertaken as described in McClelland et al. (2016). Briefly, genomic DNA was extracted from fin samples for each of the T and NT fish selected for analysis (as described above) using a DNeasy kit (Qiagen, Germany) following the manufacturer’s instructions. DNA sequencing and SNP discovery were performed at the Institute for Genomic Diversity at Cornell University using their discovery pipeline implemented in TASSEL v. 3 [75, 76]. DNA was digested with the restriction enzyme EcoT221 and strands were identified using individual-specific barcodes. Tags with fewer than 10 reads per tag were removed. Barcoded sequences were combined into a list of unique sequence tags. These tags were then aligned to the coho salmon reference genome (NCBI Assembly GCF_002021735.1 Okis_V1), and the genomic position of the tags was recorded. SNP discovery was performed using the aligned tags with genotypes determined by a binomial likelihood ratio method [76].

### Genome-wide association analysis

A genome-wide association (GWAS) analysis was performed using the GBS SNPs and the following phenotypes: weight, length, condition factor, and qPCR expression data for the genes described above (Table 1). Condition factor was calculated as: $CF=100\ast \frac{W}{L^3}$, where W is the weight (g) and L is the fork length (cm) [77]. A filtering approach was applied to remove spurious SNPs that had call rates < 80% and minor allele frequency (MAF) < 0.1. SNPs that did not have bi-allelic loci were also removed as were individuals with more than 50% missing data. To minimize the presence of duplicated loci and error prone loci, loci with Mendelian errors greater than 5% were excluded using PLINK [78]. Association analyses were performed using TASSEL v. 5 [79] with sex as a covariate. Associations between SNP markers and phenotypes were tested using a general linear model (GLM) in TASSEL with the default settings. TASSEL analyses were performed using the complete data set (results not shown since the transgenotype dominates effects on phenotype) and for subsets comprised of transgenic-only (T) and non-transgenic-only (NT) fish. Significant associations were determined using a false discovery rate (FDR) of 0.05 [80]. Venn diagrams summarizing significant findings were generated using the online tool available at http://bioinformatics.psb.ugent.be/webtools/Venn/.

## Supplementary information

**Additional file 1: Table S1.** Genes differentially expressed between large transgenic (T_Large_) and non-transgenic (NT_Large_) fish with a ≥ 3 fold change in expression level. Negative log2Ratios indicate genes that had higher expression levels in NT_Large_ while positive values are genes with higher expression levels in T_Large_. **Table S2.** Genes differentially expressed between small transgenic (T_Small_) and non-transgenic (NT_Small_) fish with a ≥ 3 fold change in expression level. Negative log2Ratios indicate genes that had higher expression levels in NT_Small_ while positive values are genes with higher expression levels in T_Small_. **Table S3.** Genes differentially expressed between large (T_Large_) and small (T_Small_) transgenic (T) fish with a ≥ 3 fold change in expression level. Negative log2Ratios indicate genes that had higher expression levels in T_Large_ while positive values are genes with higher expression levels in T_Small_. **Table S4.** Genes differentially expressed between large (NT_Large_) and small non-transgenic (NT_Small_) fish with a ≥ 3 fold change in expression level. Negative log2Ratios indicate genes that had higher expression levels in NT_Large_ while positive values are genes with higher expression levels in NT_Small_. **Table S5.** Gene Ontology (GO) Biological Process categories for differentially expressed genes (DEGs) identified in comparisons between transgenotypes within size groups (large non-transgenic, NT_Large_; large transgenic, T_Large_; small non-transgenic, NT_Small_; and small transgenic, T_Small_). The total number of DEGs found in GO categories in the whole genome are given as well as observed (obs) and expected (exp) GO terms represented in the study dataset. The c^2^ value for GO terms where the observed number of DEGs differed significantly from expected (*p* < 0.05) is given (c^2^), along with deviations (dev) from expected as observed - expected, and the relative percent (rel %) of the genome represented by that GO-term. **Table S6.** Gene Ontology (GO) Biological Process categories for the differentially expressed genes identified in comparisons between size groups within transgenotypes (large non-transgenic, NT_Large_; large transgenic, T_Large_; small non-transgenic, NT_Small_; and small transgenic, T_Small_). The total number of DEGs found in GO categories in the whole genome are given as well as observed (obs) and expected (exp) GO terms represented in the study dataset. The c^2^ value for GO terms where the observed number of DEGs differed significantly from expected (*p* < 0.05) is given (c^2^), along with deviations (dev) from expected as observed - expected, and the relative percent (rel %) of the genome represented by that GO-term. **Table S7.** SNP markers that were significantly associated with expression levels of assessed genes or phenotypic traits in different size groups of either Transgenic (T) or Non-Transgenic (NT) fish. *P*-values from the GLM analysis are given along with the adjusted q value (FDR = 0.05); significant q values are shown in bold. Traits are as for Table 1; linkage groups (LG) with NCBI Reference numbers (REF), and position (POS) on the linkage group are given, along with the Major and Minor alleles for each SNP. a: SNP marker associated with multiple traits; b: the same marker associated with different traits in T and NT; c: the same marker associated with different traits between size classes. **Table S8.** The number of transgenic and non-transgenic coho salmon with their mean (standard deviation) weight, length and condition factor (CF) at the time of sampling. **Table S9.** Gene names and products for traits analysed by qPCR and Open Array. Oligo sequences of forward (F) and reverse (R) primers and probes are given along with gene locations and NCBI transcriptome IDs (for Open Array genes). General functions are indicated.

**Additional file 2:** **Figure S1.** Gene Ontology (GO) Biological Process categories for the differentially expressed genes (DEGs) identified in comparisons between transgenotypes (transgenic fish, T, and non-transgenic fish, NT) for large and small fish. **Figure S2.** Box plots represent the median and 25% quantiles for relative gene expression for large and small transgenic (TLarge; TSmall) and nontransgenic (NT Large and NTSmall) fish. Groups with the different letters are significantly different (Tukey HSD, *p* < 0.05). Gene abbreviations are as for Table S2. **Figure S3.** Distribution of SNPs across Coho Salmon linkage groups. **Figure S4.** Histogram of lengths (cm) for transgenic (T) and non-transgenic (NT) fish at the time of sampling. A subset of these fish were used for further analysis as described in the text. **Figure S5.** Manhattan plots of SNP number and linkage group (1–30, and unassigned (U)) for expression traits in transgenic (T) and non-transgenic (NT) fish. Significant SNPs (FDR = 0.05) are indicated by red triangles; dotted line indicates significant q value. Name abbreviations are as for Table S2. **Figure S6.** Manhattan plots of SNP number and linkage group (1–30, and unassigned (U)) for weight, length and condition factor in transgenic (T) and non-transgenic (NT) fish. Significant SNPs (FDR = 0.05) are indicated by red triangles; the dotted line indicates the significant q value.

## Data Availability

The datasets generated and/or analysed during the current study are available from the following locations: RNAseq and GBS data have been submitted to the NCBI BioProject database (https://www.ncbi.nlm.nih.gov/bioproject; accession: PRJNA597081); coho salmon whole genome sequence data used for GO annotation is also available in the NCBI GenBank database (accession: GDQG00000000.1); differentially expressed genes are given in Tables S1, S2, S3, S4; GO terms are given in Tables S5, S6.

## Abbreviations

ACTB: β-actin
AGBL5: Cytosolic carboxypeptidase-like protein 5
ALB: Albumin
AST: Aspartate aminotransferase
C3–4: Complement component C3–4
CF: Condition factor
CPT1: Carnitine palmitoyltransferase 1b
DEGs: Differentially expressed genes
ELA1: Elastase 1
FAD6: Fatty acid desaturase 6
FAS: Fatty acid synthase medium chain
FBP: Fructose 1,6 biphosphatase
FDR: False discovery rate
G6PASE1: Glucose-6-phosphatase
G6PDH: Glucose 6-phosphate dehydrogenase
GBS: Genotype by sequencing
GH: Growth hormone
GHR: Growth hormone receptor
GLDH: Glutamate dehydrogenase
GLK: Glucokinase
GLM: General linear model
GLUT1: Glucose transporter 1
GOGAT: Glutamine synthetase
GWAS: Genome-wide association study
HK1: Hexokinase 1
HOAD: Hydroxyacyl dehydrogenase
IGF1: Insulin-like growth factor I
IGF1R: Insulin-like growth factor I receptor
IGFBP1: Insulin-like growth factor binding protein I
IGFBP2B2: Insulin-like growth factor binding protein 2B2
IGF-I: Insulin-like growth factor
IGFII: Insulin-like growth factor II
MAF: Minor allele frequency
MID1IP1: Mid1-interacting protein
NT: Non-transgenic
PEPCK: Phosphoenolpyruvate carboxy kinase
PLCE1: 1-phosphatidylinositol-4,5-bisphosphate phosphodiesterase ε-1
qPCR: Quantitative polymerase chain reaction
QTL: Quantitative trait loci
SNP: Single nucleotide polymorphism
T: Transgenic
TRB: Thyroid hormone receptor b
UBQ: Ubiquitin
X6PFK: 6-phosphofructokinase
