# Supplementary material for: Loci associated with variation in gene expression and growth in juvenile salmon are influenced by the presence of a growth hormone transgene
Source: BMC Genomics. 2020 Feb 27;21:185. doi: 10.1186/s12864-020-6586-0 (PMC7045383; doi:10.1186/s12864-020-6586-0)

Figure S1: Gene Ontology (GO) Biological Process categories for the differentially expressed genes (DEGs) identified in comparisons between transgenotypes (transgenic fish, T, and non-transgenic fish, NT) for large and small fish. SEE EXCEL FILE

Figure S2: Box plots represent the median and 25% quantiles for relative gene expression for large and small transgenic (TLarge; TSmall) and nontransgenic (NT Large and NTSmall) fish. Groups with the different letters are significantly different (Tukey HSD, p<0.05). Gene abbreviations are as for Table S2.

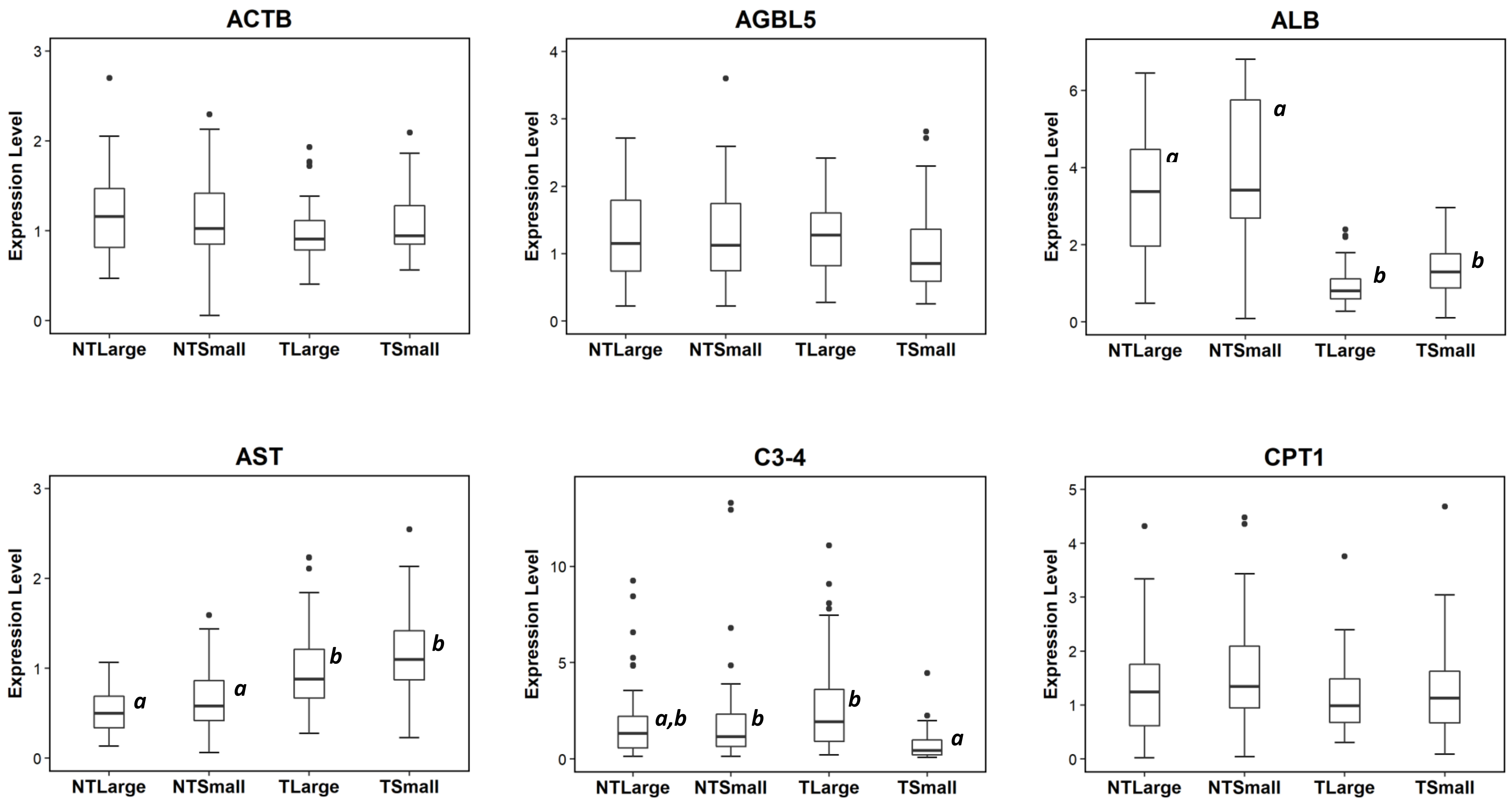

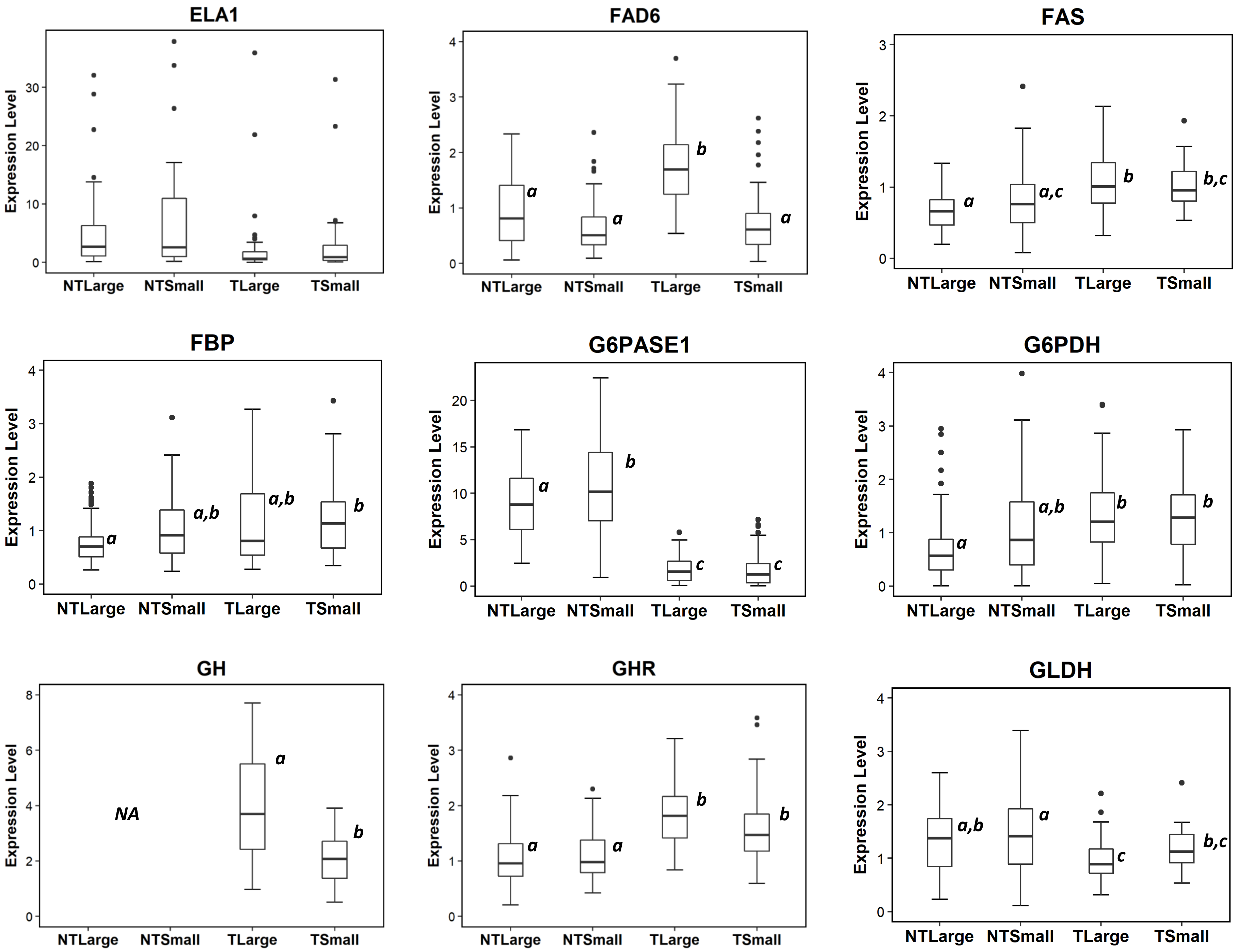
ELA1
FAD6
FAS
FBP
G6PASE1
G6PDH
GH
GHR
GLDH
Expression Level
NTLarge
NTSmall
TLarge
TSmall
NA
a
b
c
a,b
a,c
b,c

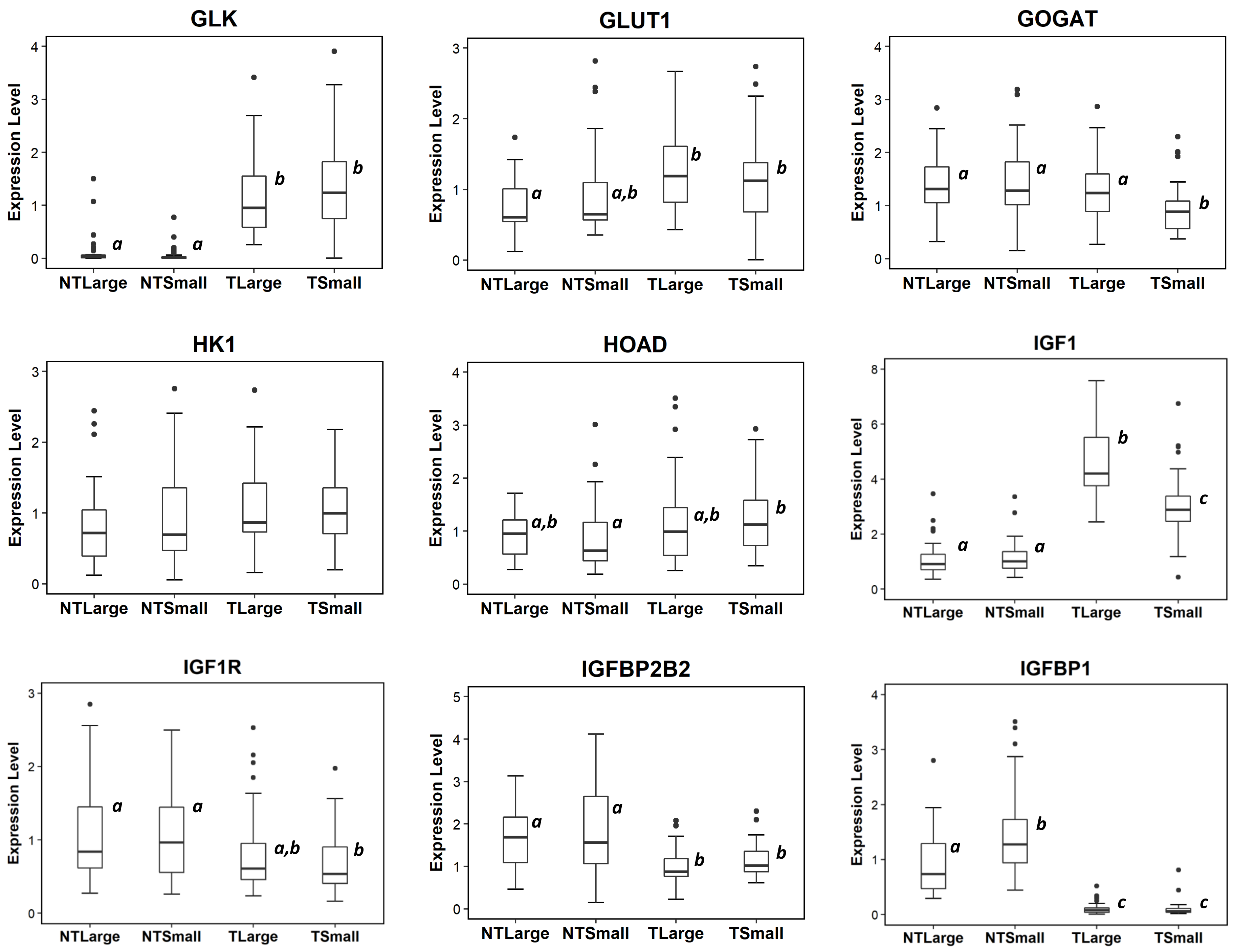
GLK
Expression Level
0 1 2 3 4
a a b b
NTLarge NTSmall TLarge TSmall
GLUT1
Expression Level
0 1 2 3
a a,b b b
NTLarge NTSmall TLarge TSmall
GOGAT
Expression Level
0 1 2 3 4
a a a b
NTLarge NTSmall TLarge TSmall
HK1
Expression Level
0 1 2 3
NTLarge NTSmall TLarge TSmall
HOAD
Expression Level
0 1 2 3 4
a,b a a,b b
NTLarge NTSmall TLarge TSmall
IGF1
Expression Level
0 2 4 6 8
a a b c
NTLarge NTSmall TLarge TSmall
IGF1R
Expression Level
0 1 2 3
a a a,b b
NTLarge NTSmall TLarge TSmall
IGFBP2B2
Expression Level
0 1 2 3 4 5
a a b b
NTLarge NTSmall TLarge TSmall
IGFBP1
Expression Level
0 1 2 3 4
a b c c
NTLarge NTSmall TLarge TSmall

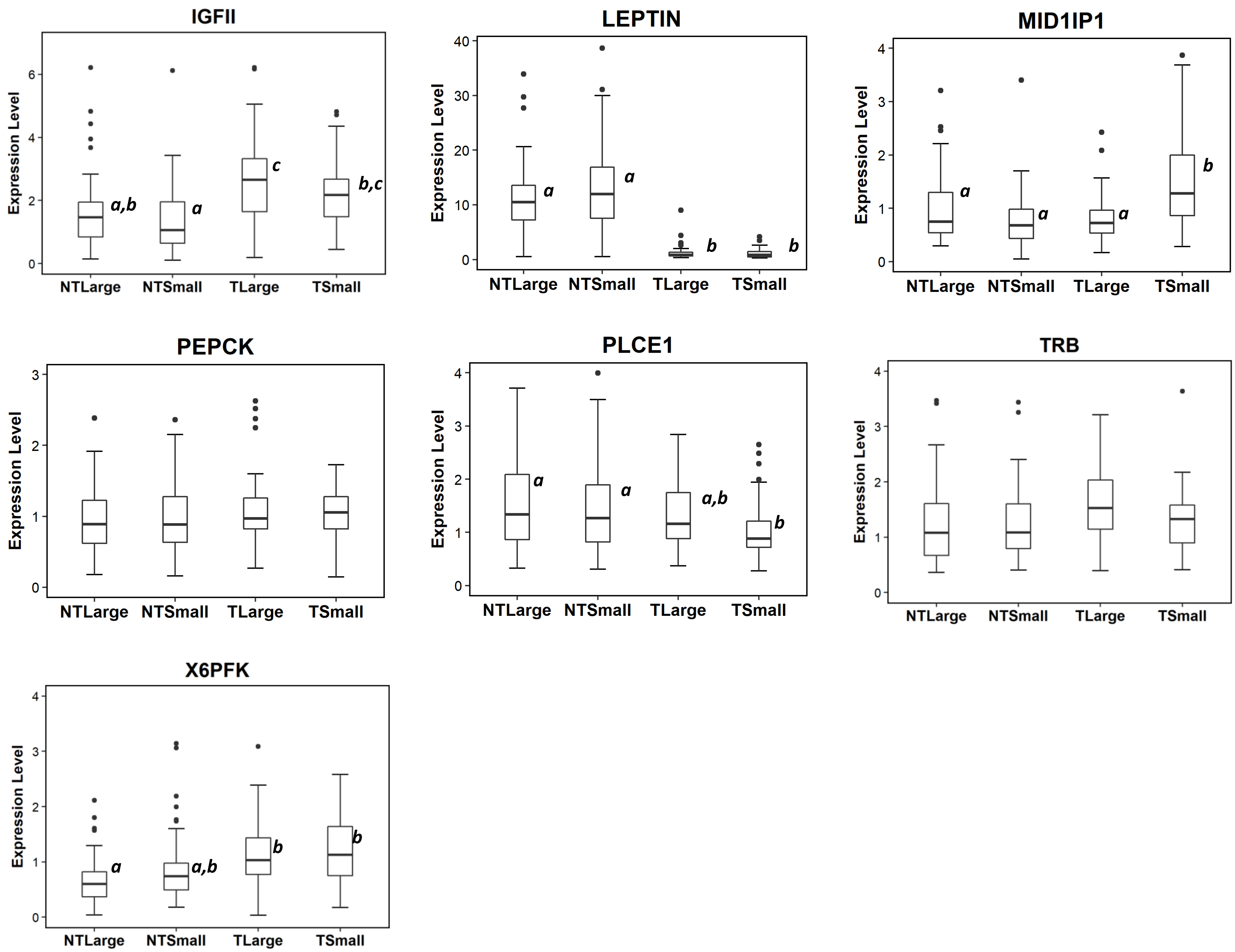
IGFII
Expression Level
0
2
4
6
NTLarge
NTSmall
TLarge
TSmall
a,b
a
c
b,c
LEPTIN
Expression Level
0
10
20
30
40
NTLarge
NTSmall
TLarge
TSmall
a
a
b
b
MID1IP1
Expression Level
0
1
2
3
4
NTLarge
NTSmall
TLarge
TSmall
a
a
a
b
PEPCK
Expression Level
0
1
2
3
NTLarge
NTSmall
TLarge
TSmall
PLCE1
Expression Level
0
1
2
3
4
NTLarge
NTSmall
TLarge
TSmall
a
a
a,b
b
TRB
Expression Level
0
1
2
3
4
NTLarge
NTSmall
TLarge
TSmall
X6PFK
Expression Level
0
1
2
3
4
NTLarge
NTSmall
TLarge
TSmall
a
a,b
b
b

Figure S3. Distribution of SNPs across Coho Salmon linkage groups.

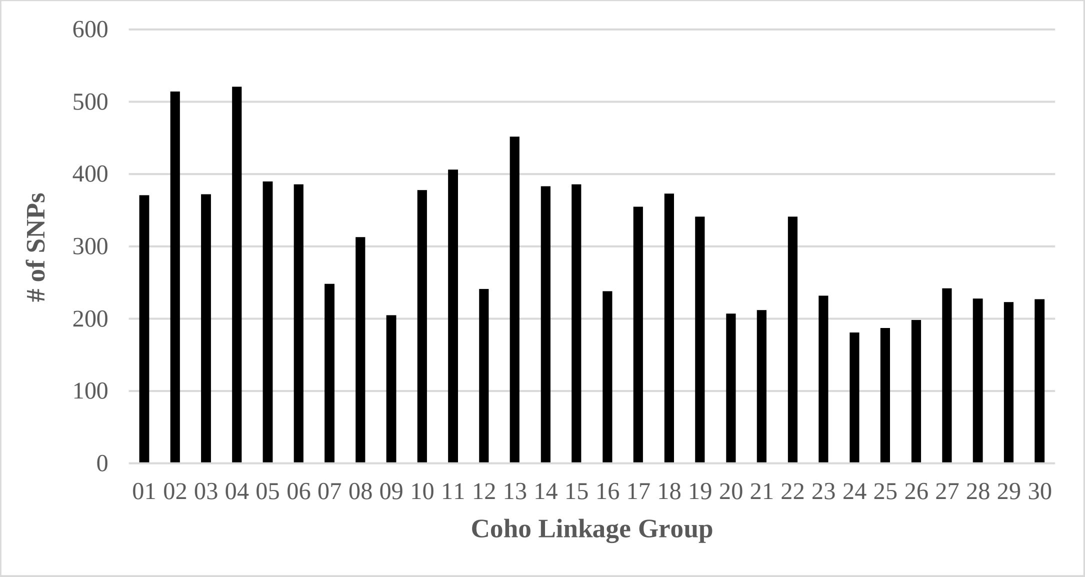

Figure S4. Histogram of lengths (cm) for transgenic (T) and non-transgenic (NT) fish at the time of sampling. A subset of these fish were used for further analysis as described in the text.

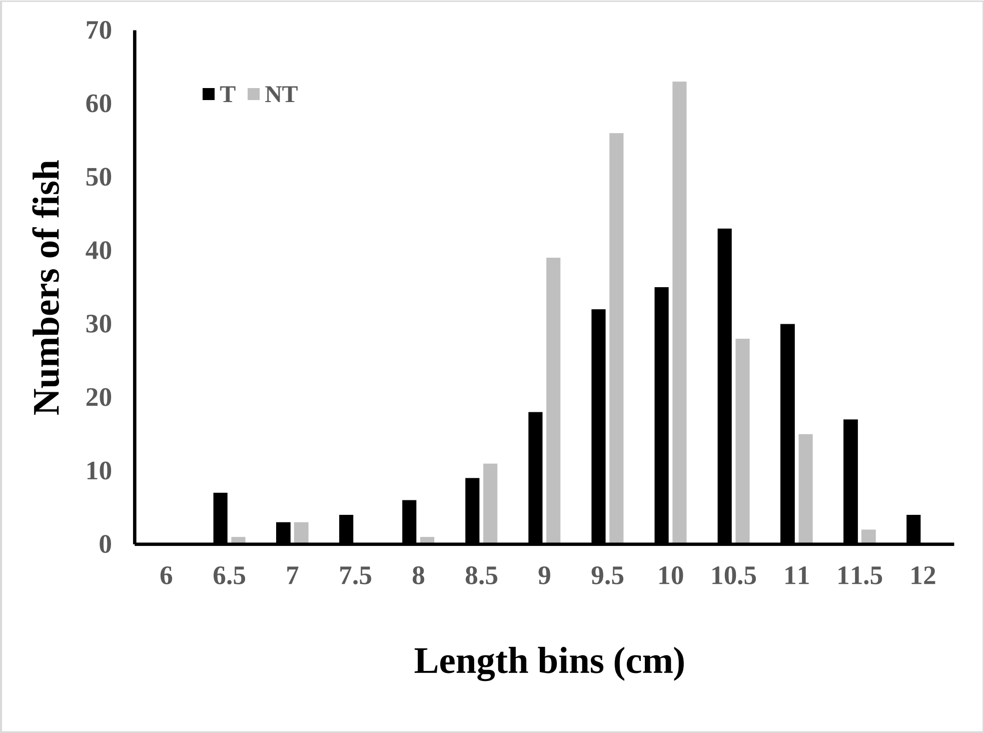

Figure S5. Manhattan plots of SNP number and linkage group (1-30, and unassigned (U)) for expression traits in transgenic (T) and non-transgenic (NT) fish. Significant SNPs (FDR=0.05) are indicated by red triangles; dotted line indicates significant q value. Name abbreviations are as for Table S2.

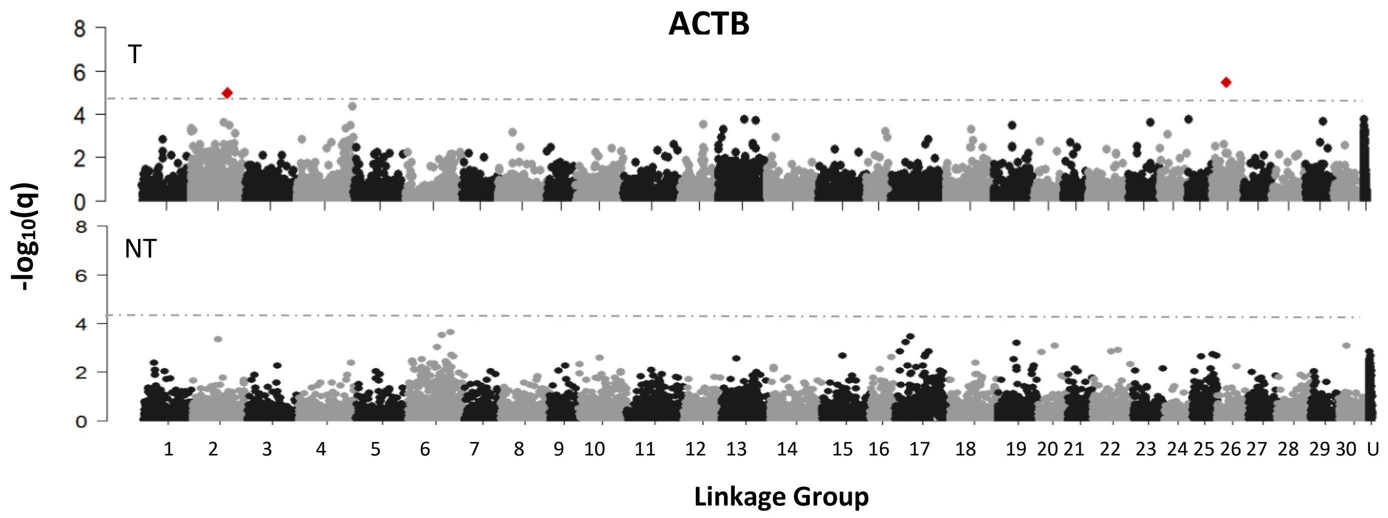

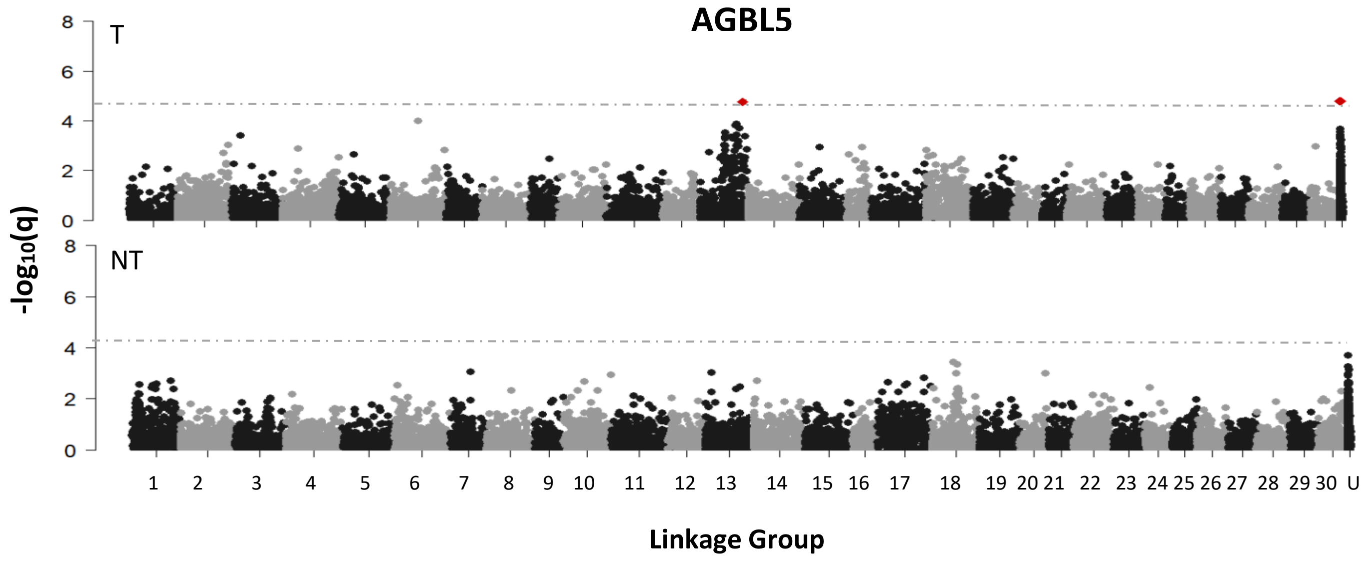

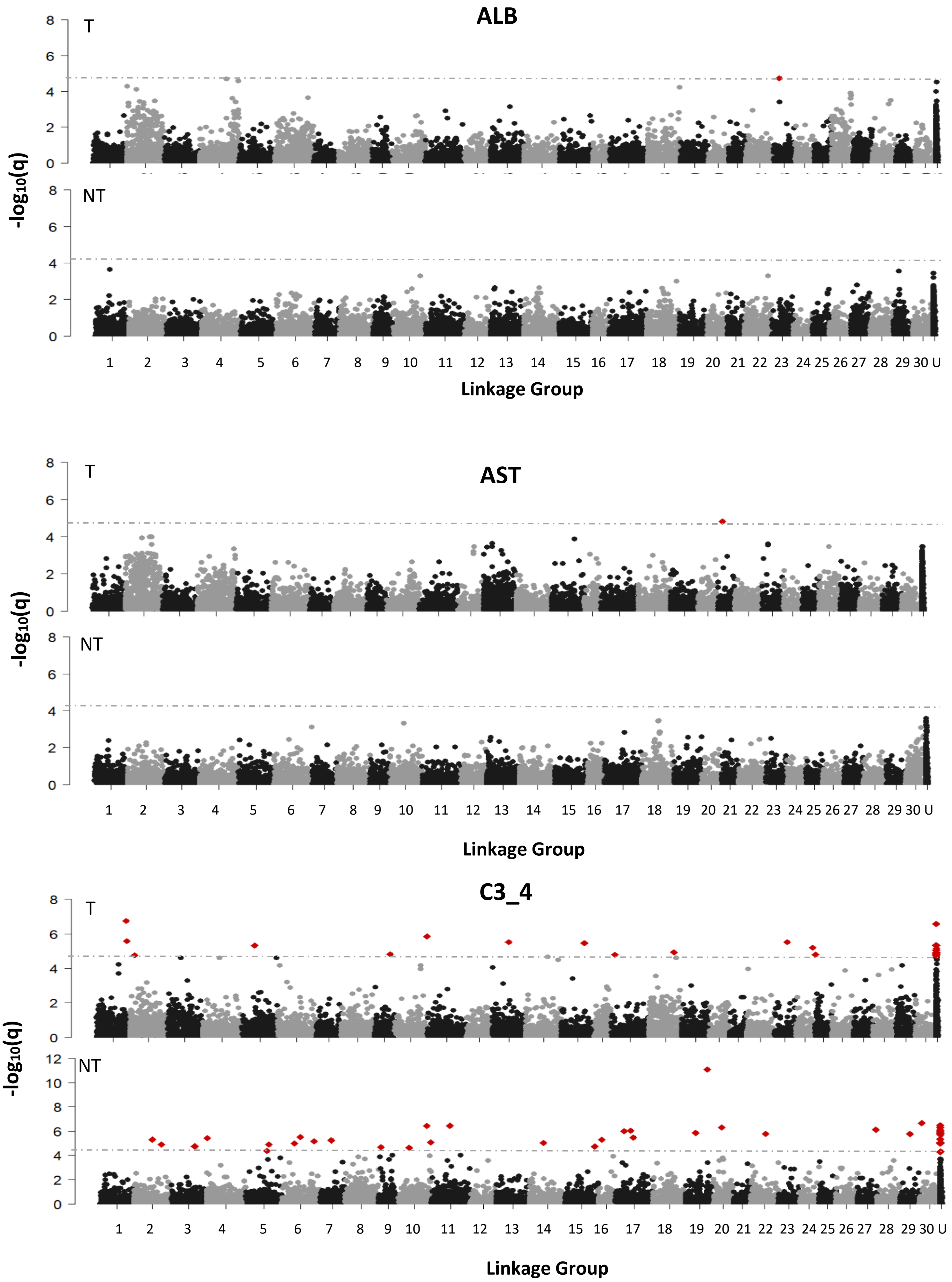
ALB
T
NT
-log10(q)
Linkage Group
1 2 3 4 5 6 7 8 9 10 11 12 13 14 15 16 17 18 19 20 21 22 23 24 25 26 27 28 29 30 U
AST
T
NT
-log10(q)
Linkage Group
1 2 3 4 5 6 7 8 9 10 11 12 13 14 15 16 17 18 19 20 21 22 23 24 25 26 27 28 29 30 U
C3_4
T
NT
-log10(q)
Linkage Group
1 2 3 4 5 6 7 8 9 10 11 12 13 14 15 16 17 18 19 20 21 22 23 24 25 26 27 28 29 30 U

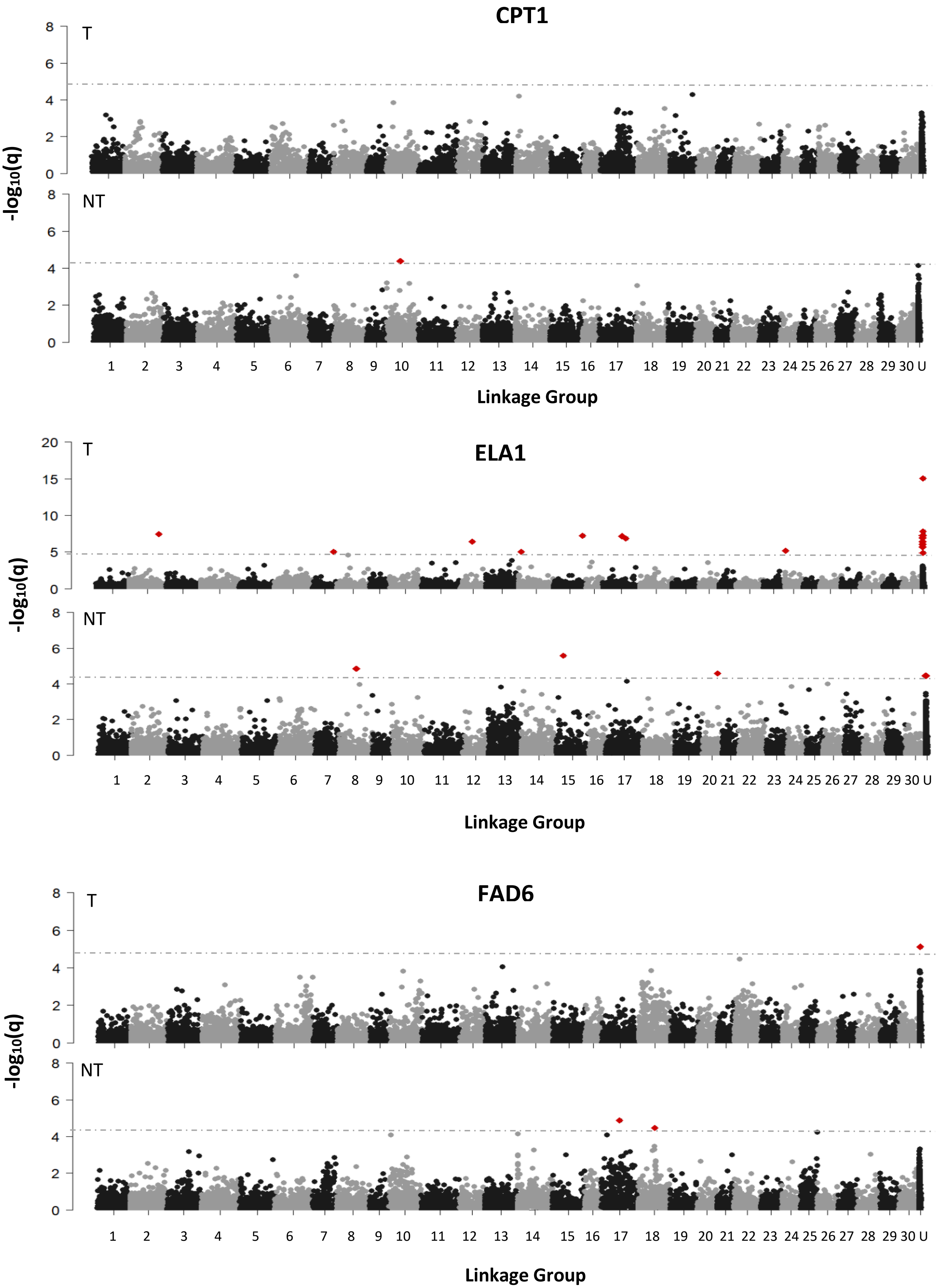
CPT1
T
NT
-log10(q)
1 2 3 4 5 6 7 8 9 10 11 12 13 14 15 16 17 18 19 20 21 22 23 24 25 26 27 28 29 30 U
Linkage Group
ELA1
T
NT
-log10(q)
1 2 3 4 5 6 7 8 9 10 11 12 13 14 15 16 17 18 19 20 21 22 23 24 25 26 27 28 29 30 U
Linkage Group
FAD6
T
NT
-log10(q)
1 2 3 4 5 6 7 8 9 10 11 12 13 14 15 16 17 18 19 20 21 22 23 24 25 26 27 28 29 30 U
Linkage Group

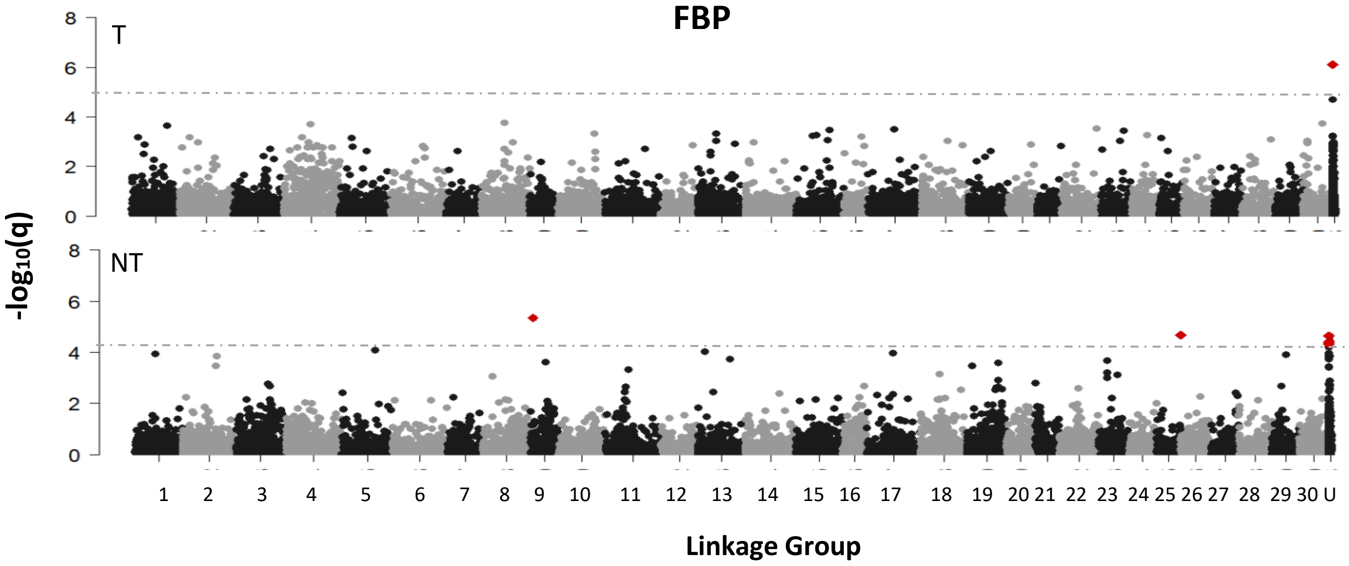
FBP
T
NT
-log10(q)
0 2 4 6 8
1 2 3 4 5 6 7 8 9 10 11 12 13 14 15 16 17 18 19 20 21 22 23 24 25 26 27 28 29 30 U
Linkage Group

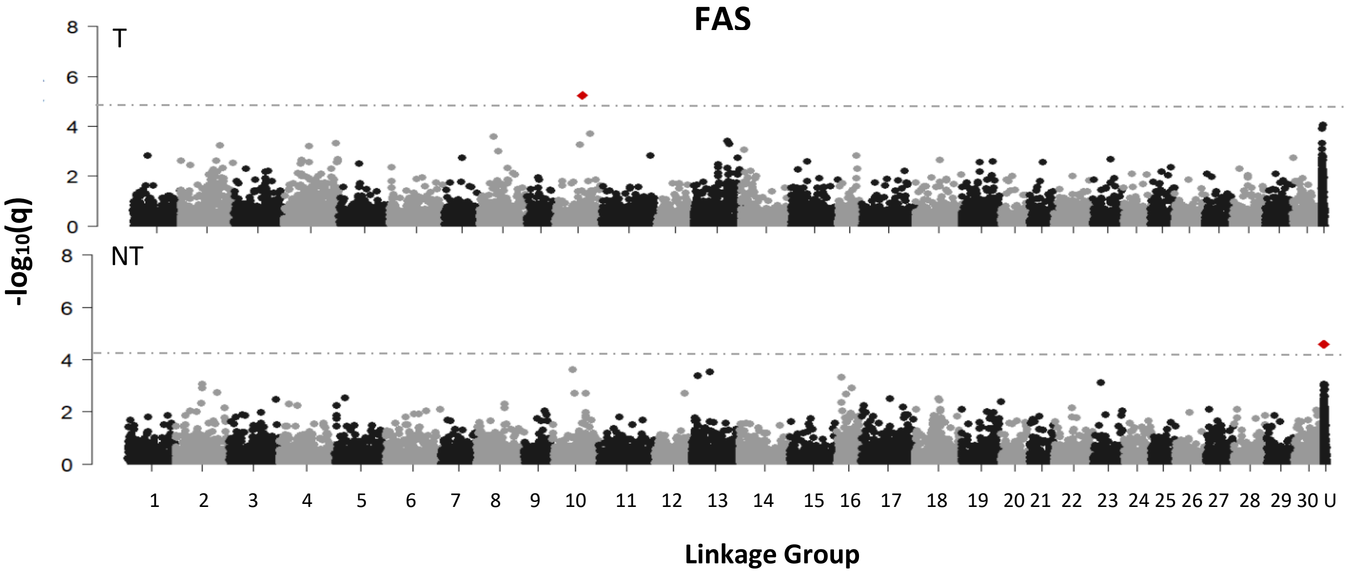
FAS
T
NT
-log10(q)
0 2 4 6 8
1 2 3 4 5 6 7 8 9 10 11 12 13 14 15 16 17 18 19 20 21 22 23 24 25 26 27 28 29 30 U
Linkage Group

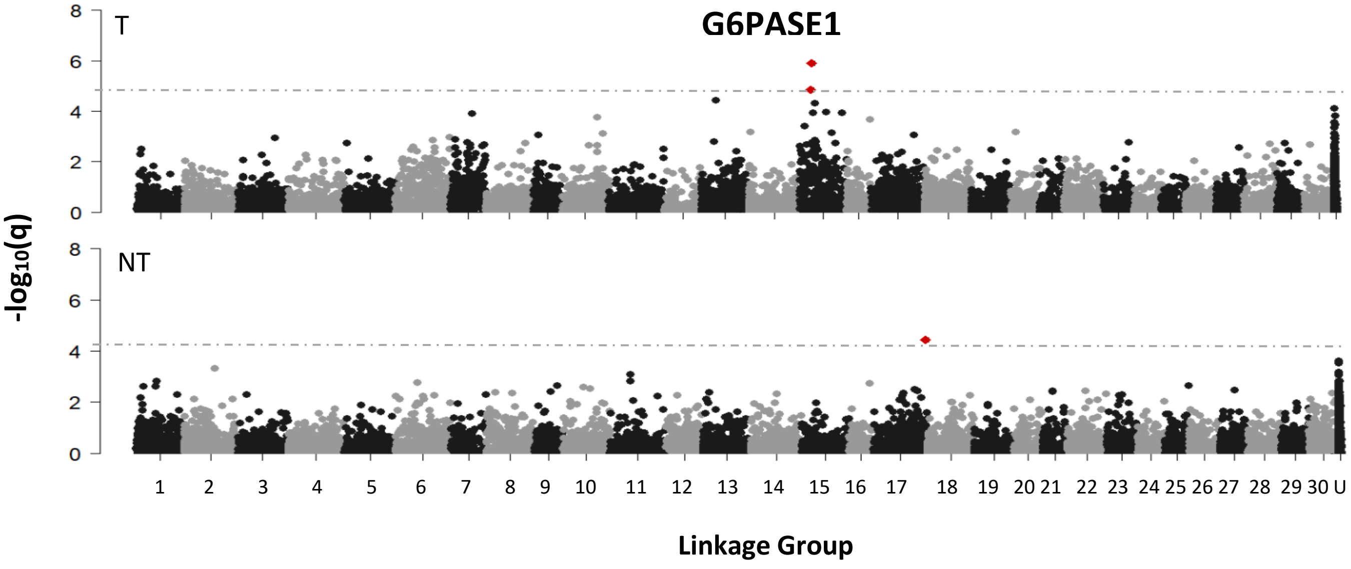
G6PASE1
T
NT
-log10(q)
0 2 4 6 8
1 2 3 4 5 6 7 8 9 10 11 12 13 14 15 16 17 18 19 20 21 22 23 24 25 26 27 28 29 30 U
Linkage Group

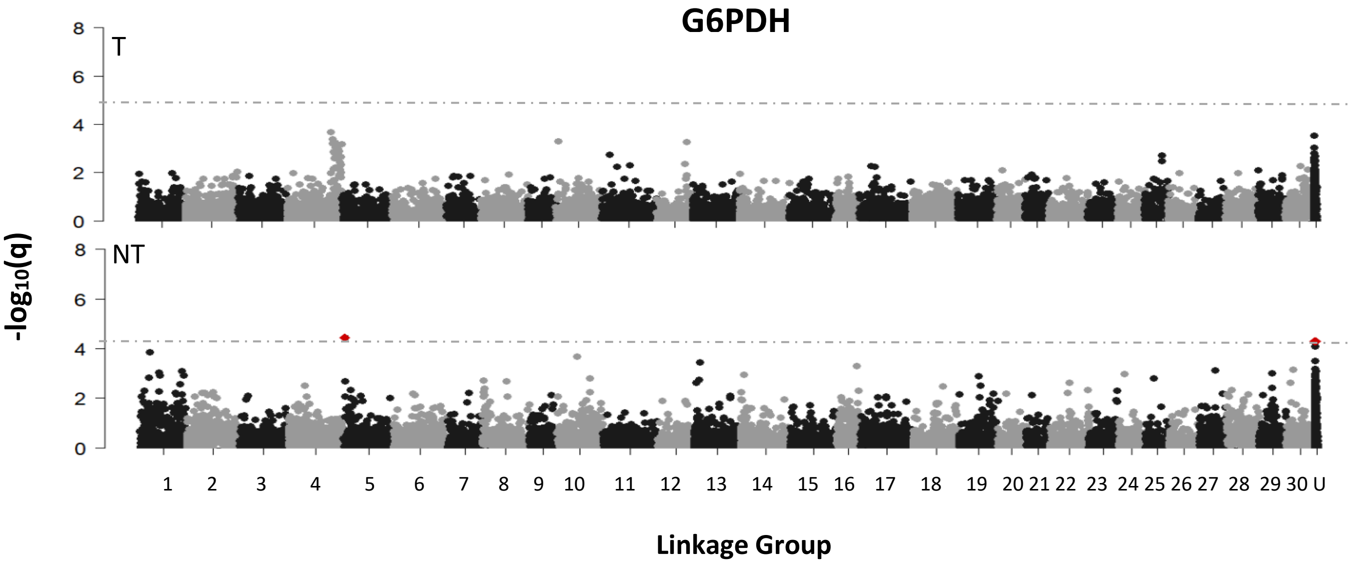
G6PDH
T
NT
-log10(q)
0
2
4
6
8
1 2 3 4 5 6 7 8 9 10 11 12 13 14 15 16 17 18 19 20 21 22 23 24 25 26 27 28 29 30 U
Linkage Group

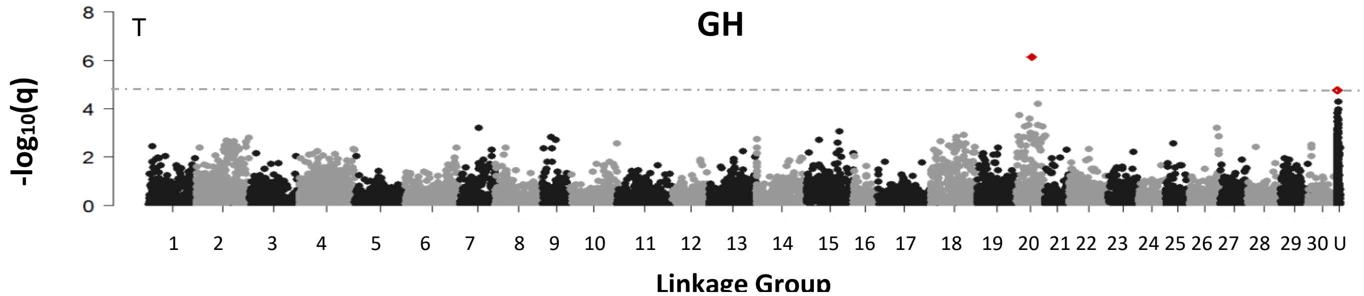
GH
T
-log10(q)
0
2
4
6
8
1 2 3 4 5 6 7 8 9 10 11 12 13 14 15 16 17 18 19 20 21 22 23 24 25 26 27 28 29 30 U
Linkage Group

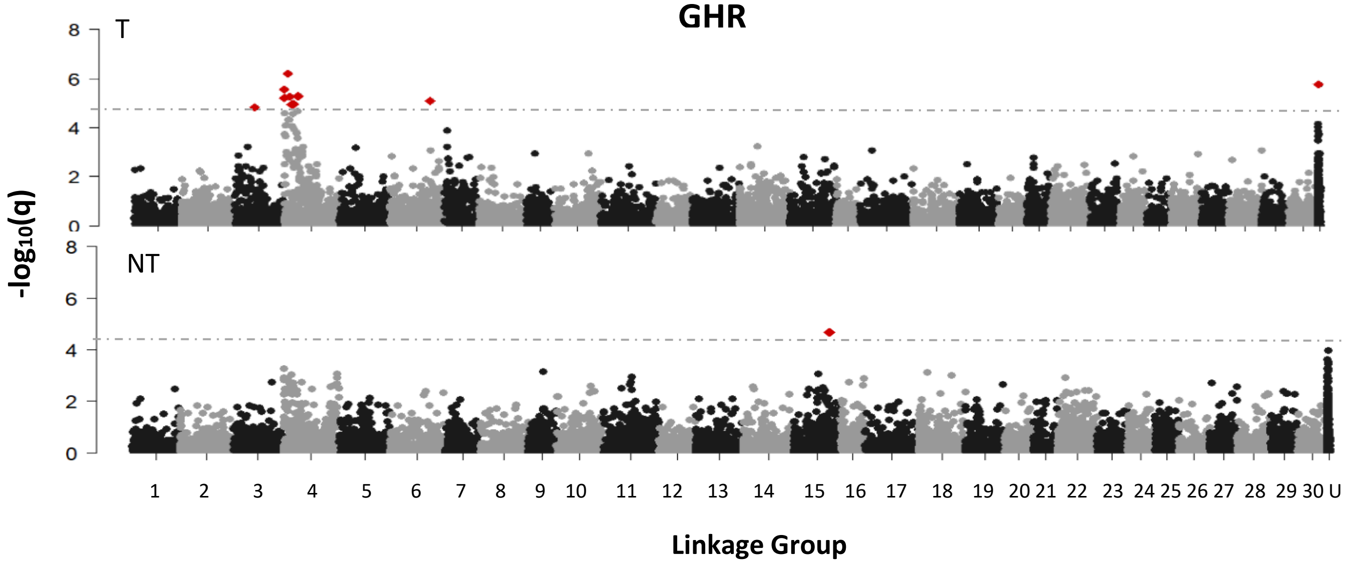
GHR
T
NT
-log10(q)
0
2
4
6
8
1 2 3 4 5 6 7 8 9 10 11 12 13 14 15 16 17 18 19 20 21 22 23 24 25 26 27 28 29 30 U
Linkage Group

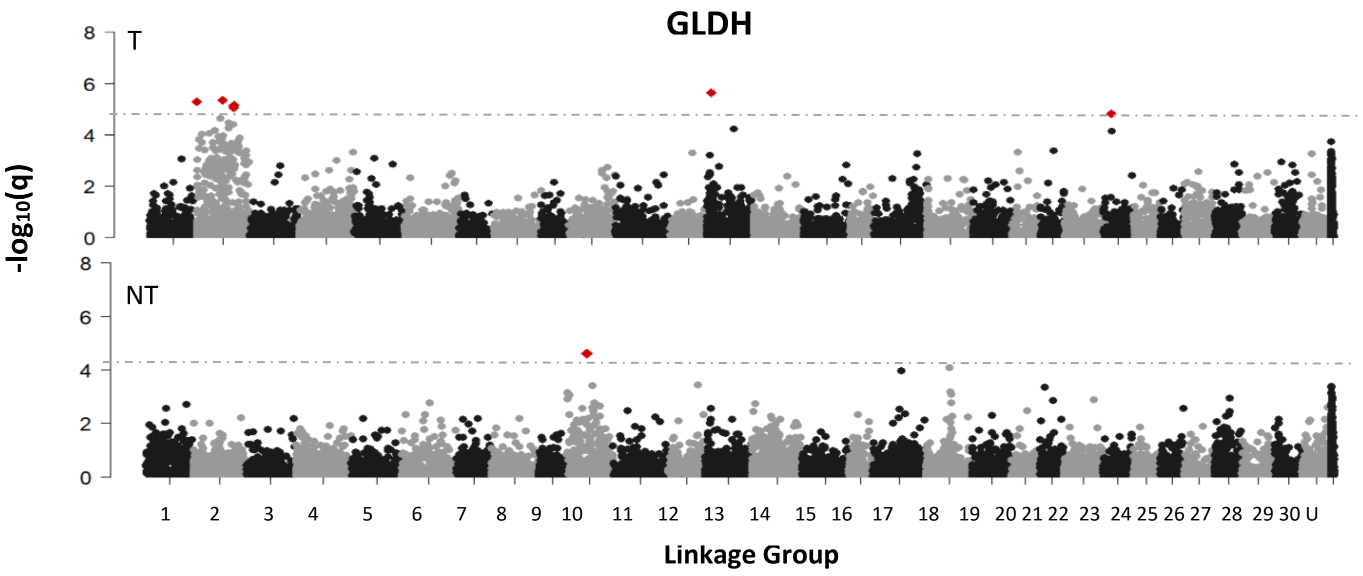
GLDH
T
NT
-log10(q)
1 2 3 4 5 6 7 8 9 10 11 12 13 14 15 16 17 18 19 20 21 22 23 24 25 26 27 28 29 30 U
Linkage Group

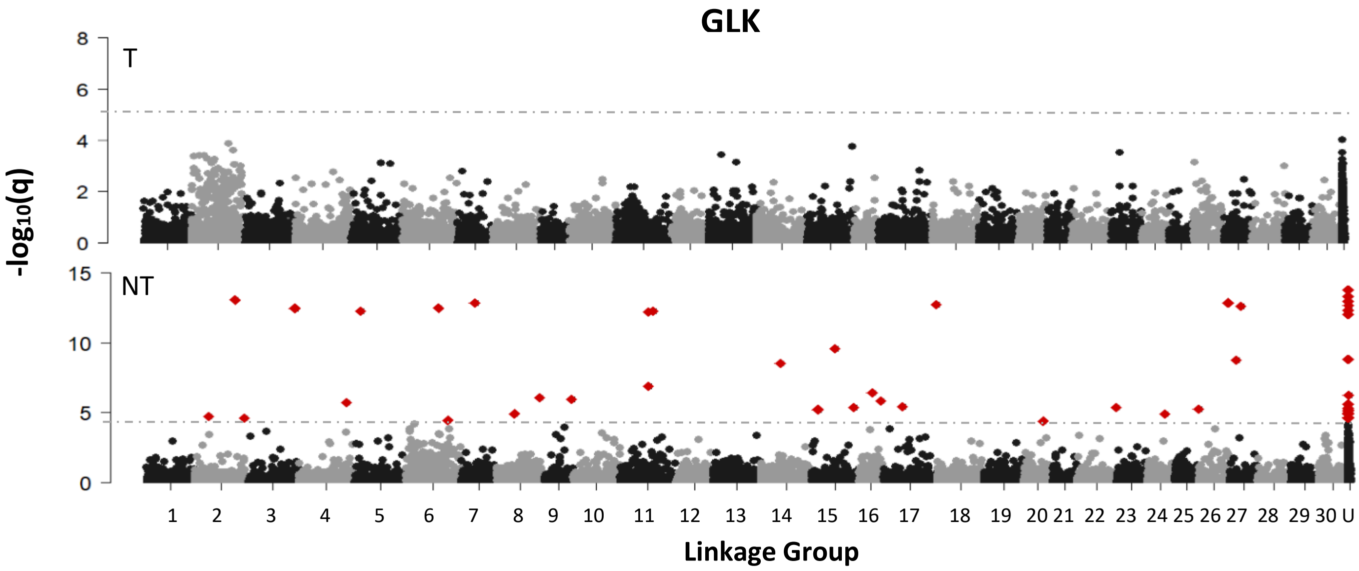
GLK
T
NT
-log10(q)
1 2 3 4 5 6 7 8 9 10 11 12 13 14 15 16 17 18 19 20 21 22 23 24 25 26 27 28 29 30 U
Linkage Group

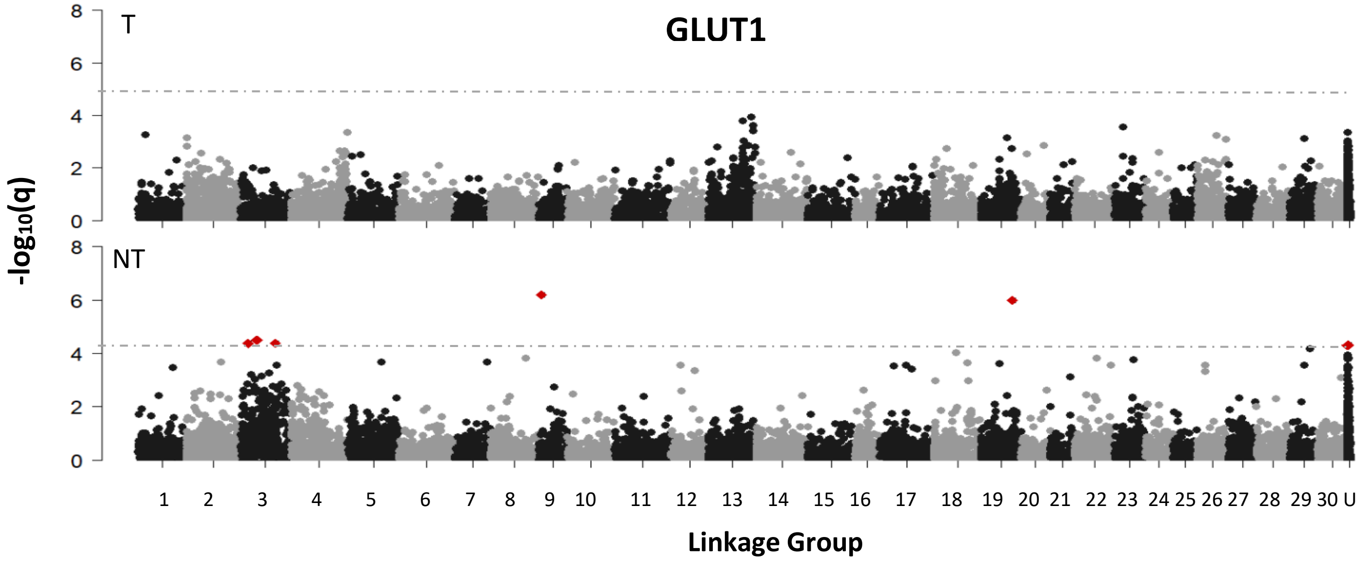
GLUT1
T
NT
-log10(q)
1 2 3 4 5 6 7 8 9 10 11 12 13 14 15 16 17 18 19 20 21 22 23 24 25 26 27 28 29 30 U
Linkage Group

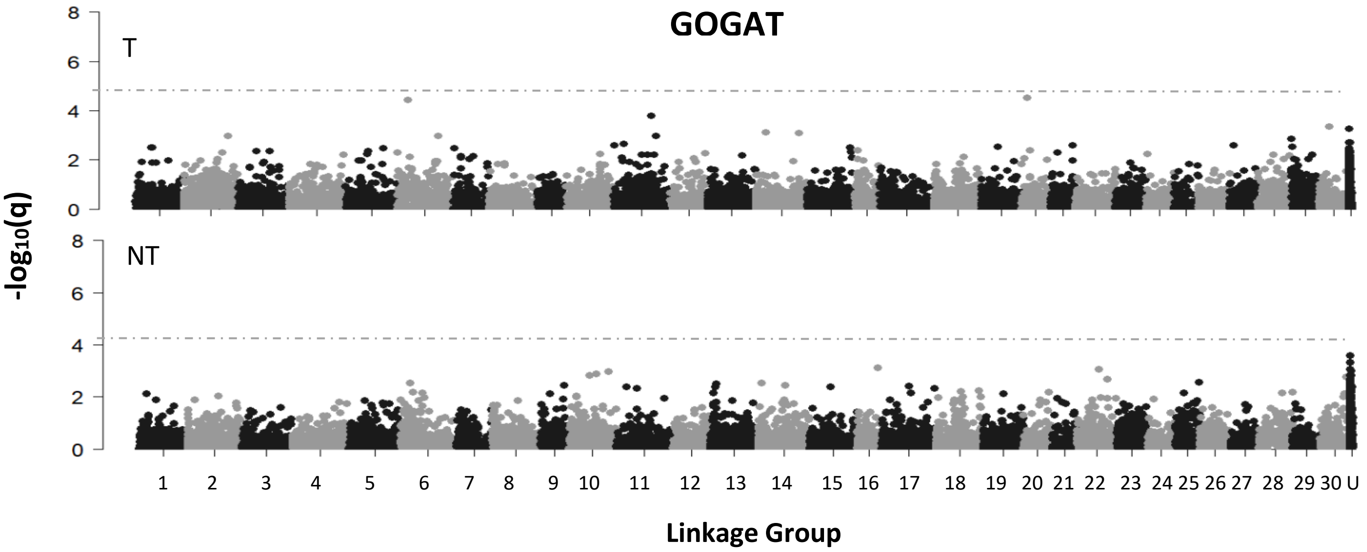
GOGAT
T
NT
-log10(q)
Linkage Group
1 2 3 4 5 6 7 8 9 10 11 12 13 14 15 16 17 18 19 20 21 22 23 24 25 26 27 28 29 30 U

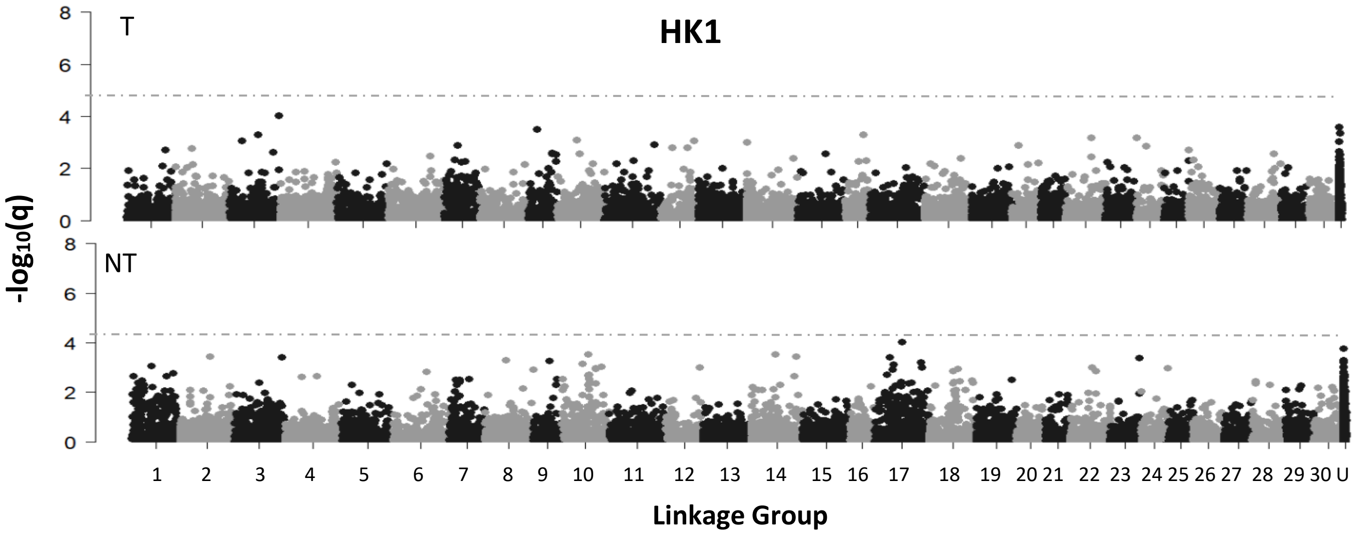
HK1
T
NT
-log10(q)
Linkage Group
1 2 3 4 5 6 7 8 9 10 11 12 13 14 15 16 17 18 19 20 21 22 23 24 25 26 27 28 29 30 U

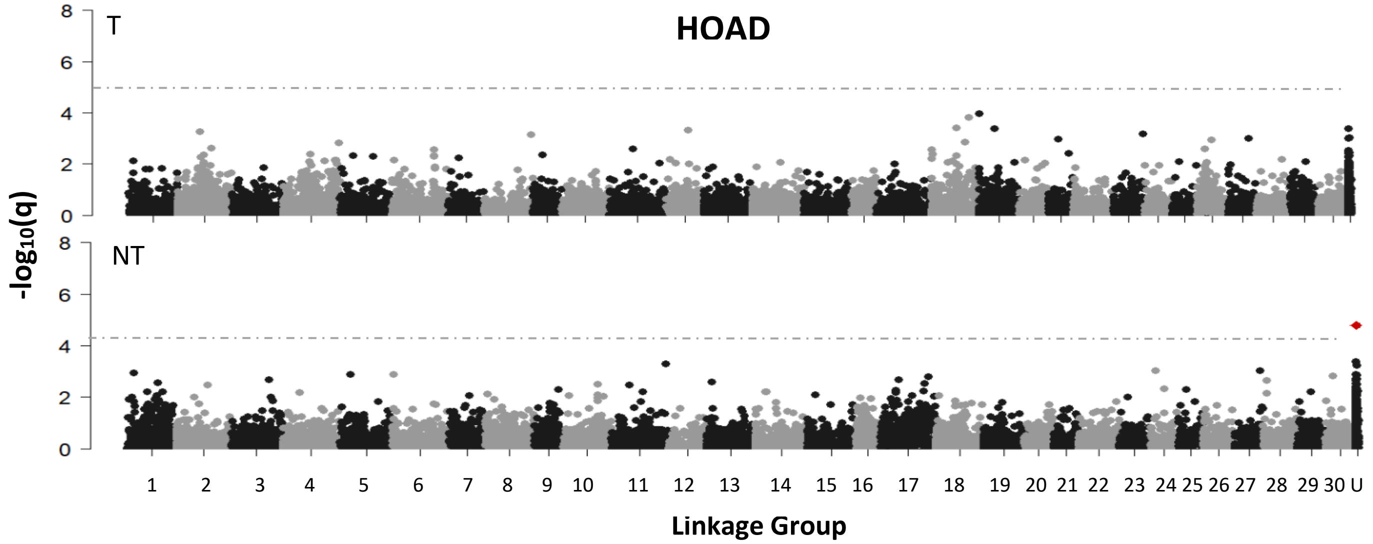
HOAD
T
NT
-log10(q)
Linkage Group
1 2 3 4 5 6 7 8 9 10 11 12 13 14 15 16 17 18 19 20 21 22 23 24 25 26 27 28 29 30 U

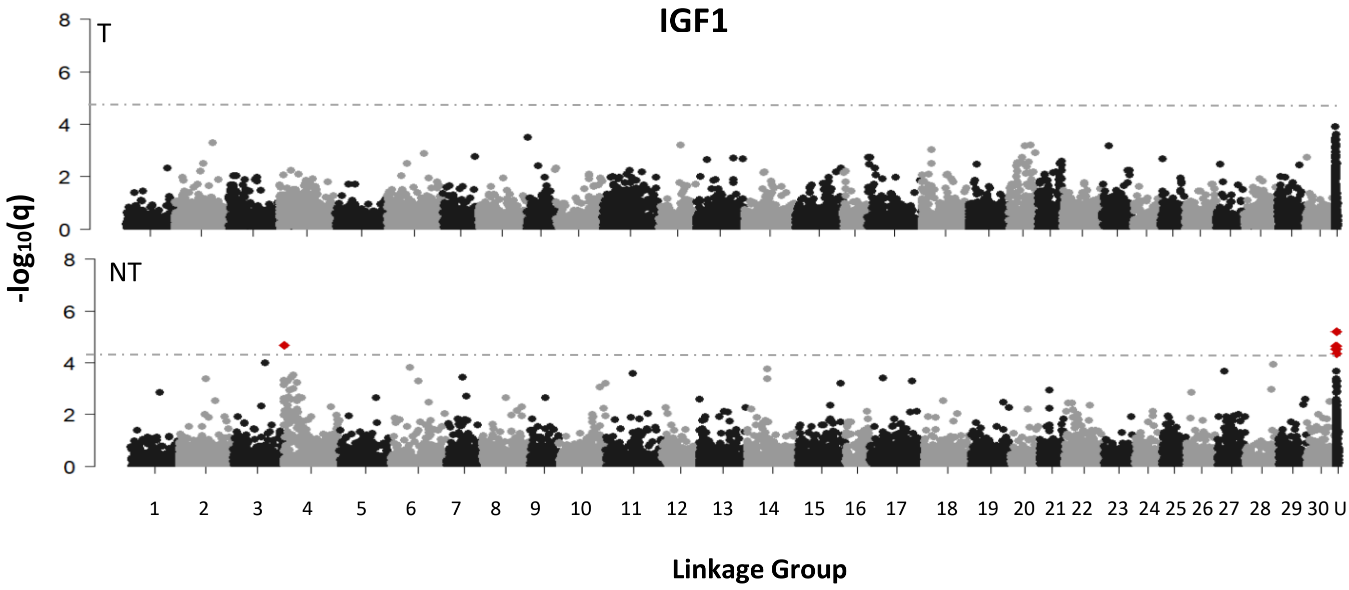
IGF1
T
NT
-log10(q)
0 2 4 6 8
1 2 3 4 5 6 7 8 9 10 11 12 13 14 15 16 17 18 19 20 21 22 23 24 25 26 27 28 29 30 U
Linkage Group

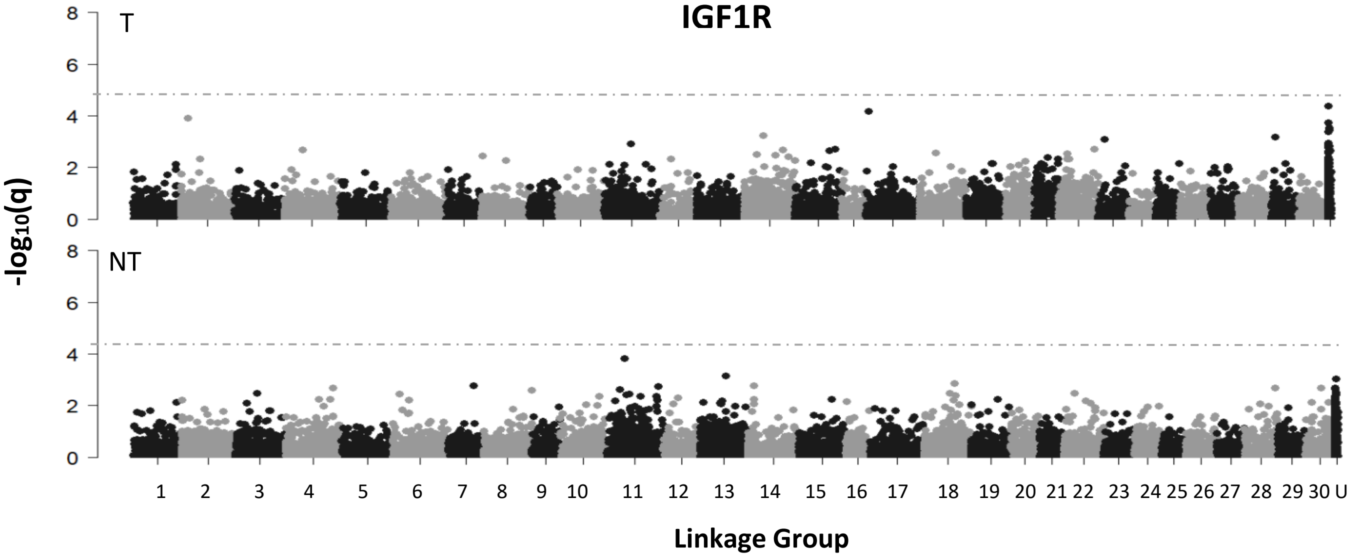
IGF1R
T
NT
-log10(q)
0 2 4 6 8
1 2 3 4 5 6 7 8 9 10 11 12 13 14 15 16 17 18 19 20 21 22 23 24 25 26 27 28 29 30 U
Linkage Group

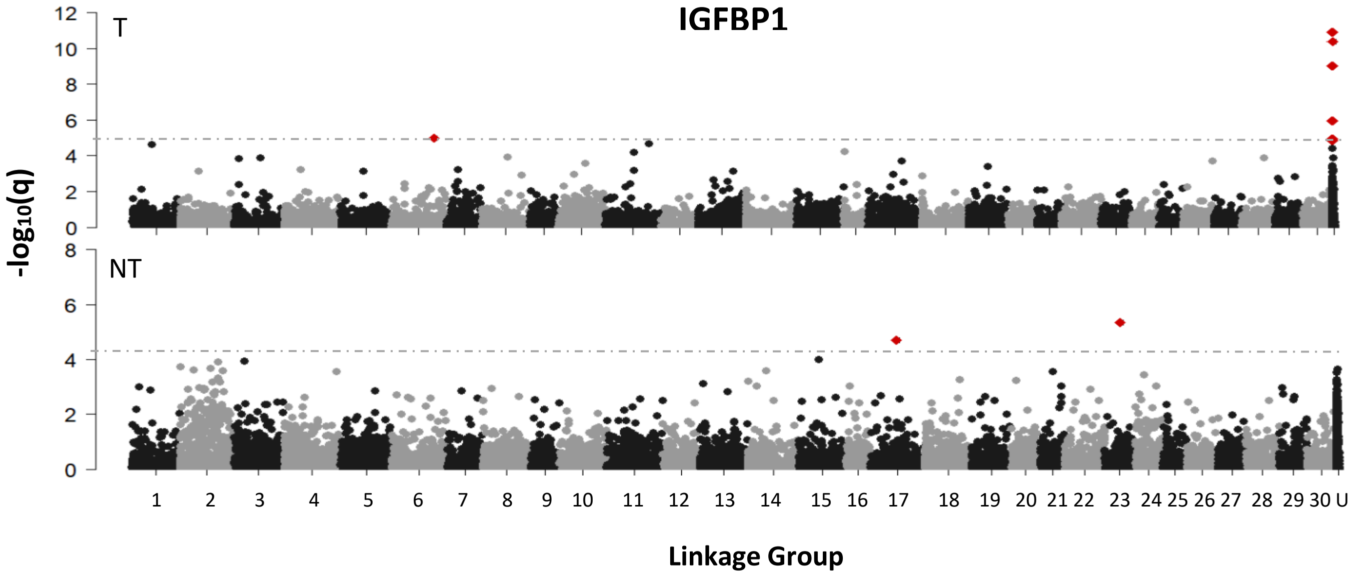
IGFBP1
T
NT
-log10(q)
0 2 4 6 8 10 12
1 2 3 4 5 6 7 8 9 10 11 12 13 14 15 16 17 18 19 20 21 22 23 24 25 26 27 28 29 30 U
Linkage Group

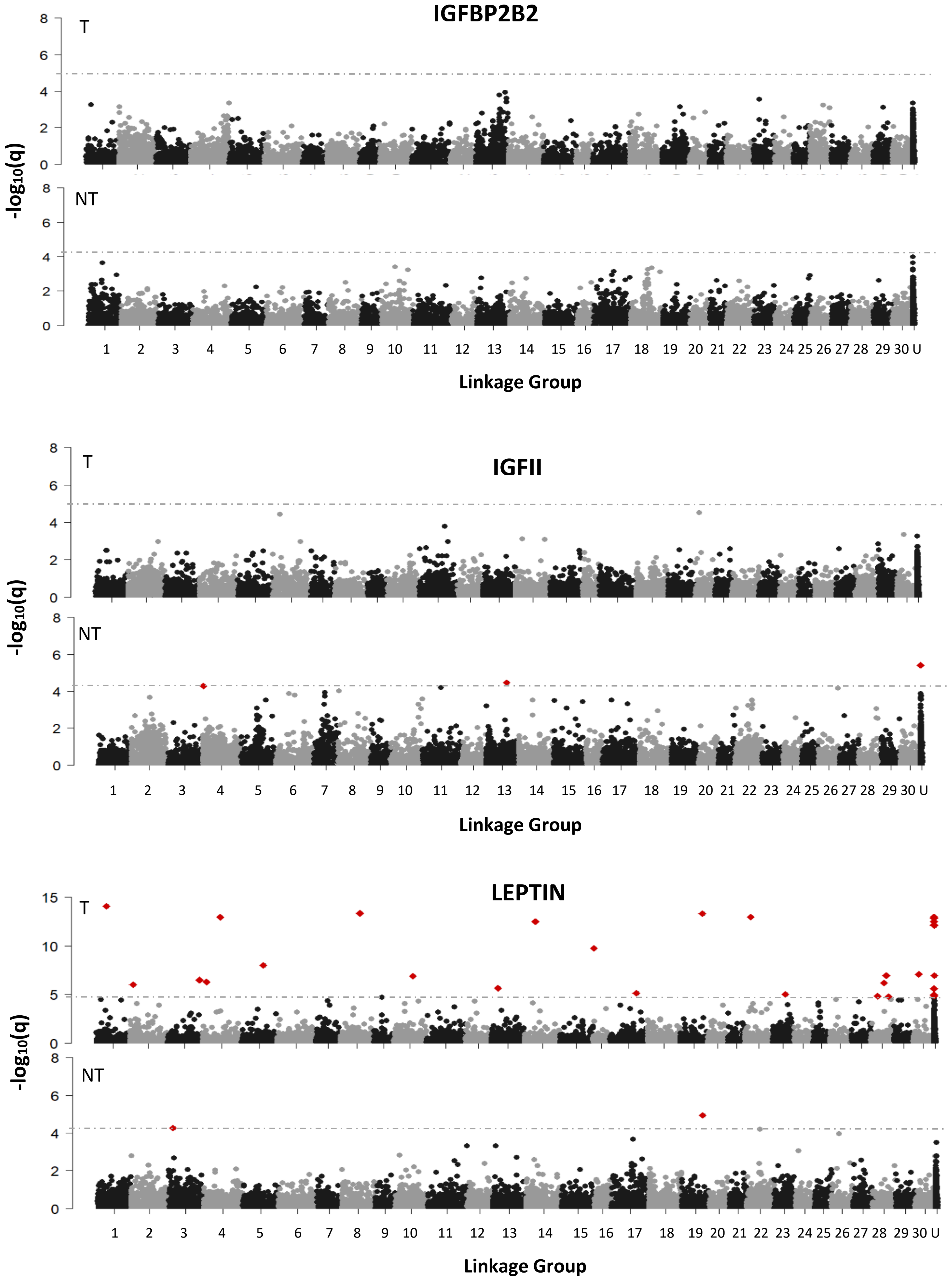
IGFBP2B2
T
NT
-log10(q)
0 2 4 6 8
1 2 3 4 5 6 7 8 9 10 11 12 13 14 15 16 17 18 19 20 21 22 23 24 25 26 27 28 29 30 U
Linkage Group
IGFII
T
NT
-log10(q)
0 2 4 6 8
1 2 3 4 5 6 7 8 9 10 11 12 13 14 15 16 17 18 19 20 21 22 23 24 25 26 27 28 29 30 U
Linkage Group
LEPTIN
T
NT
-log10(q)
0 5 10 15
0 2 4 6 8
1 2 3 4 5 6 7 8 9 10 11 12 13 14 15 16 17 18 19 20 21 22 23 24 25 26 27 28 29 30 U
Linkage Group

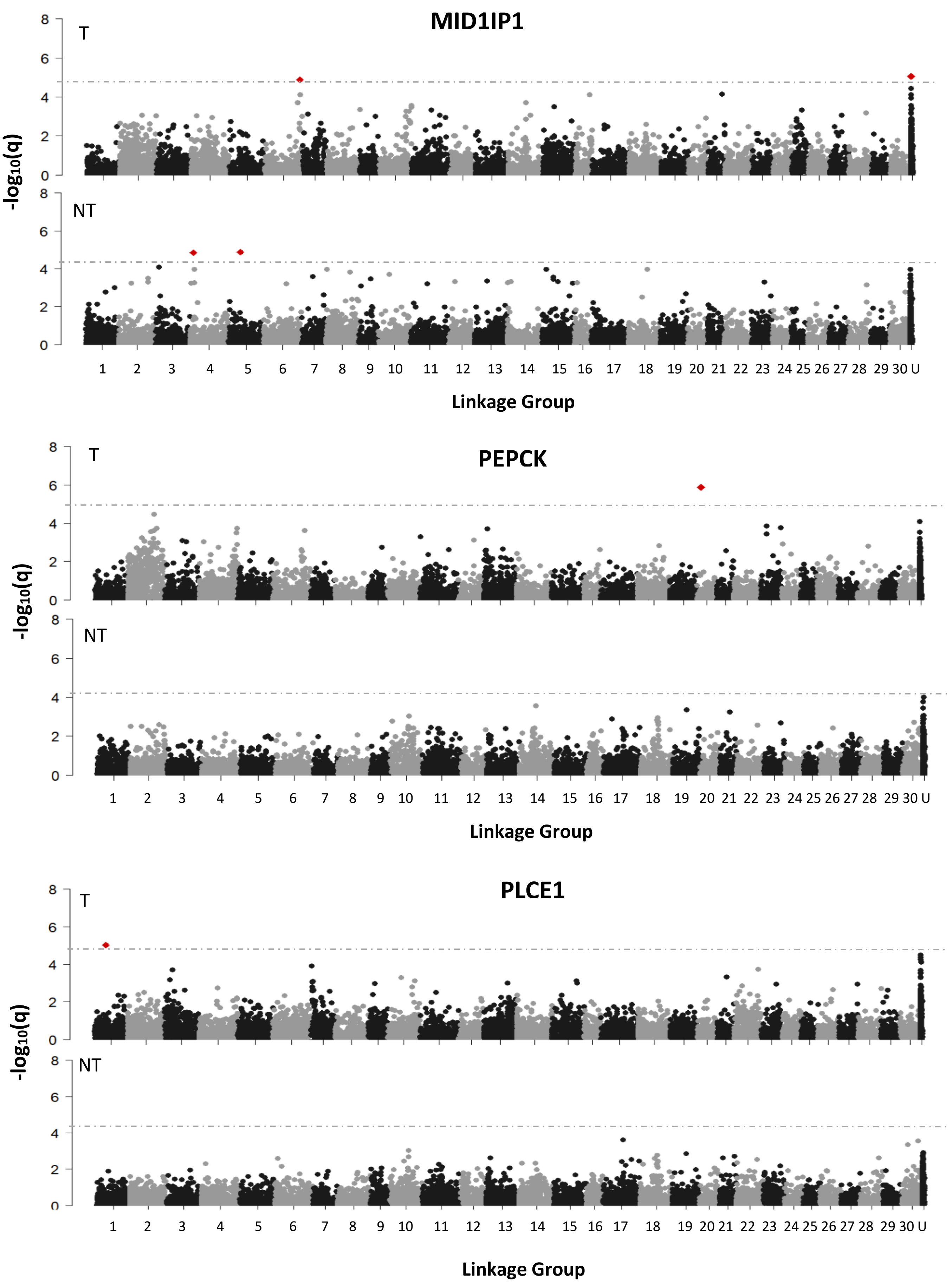
MID1IP1
T
NT
-log10(q)
Linkage Group
PEPCK
T
NT
-log10(q)
Linkage Group
PLCE1
T
NT
-log10(q)
Linkage Group
1 2 3 4 5 6 7 8 9 10 11 12 13 14 15 16 17 18 19 20 21 22 23 24 25 26 27 28 29 30 U

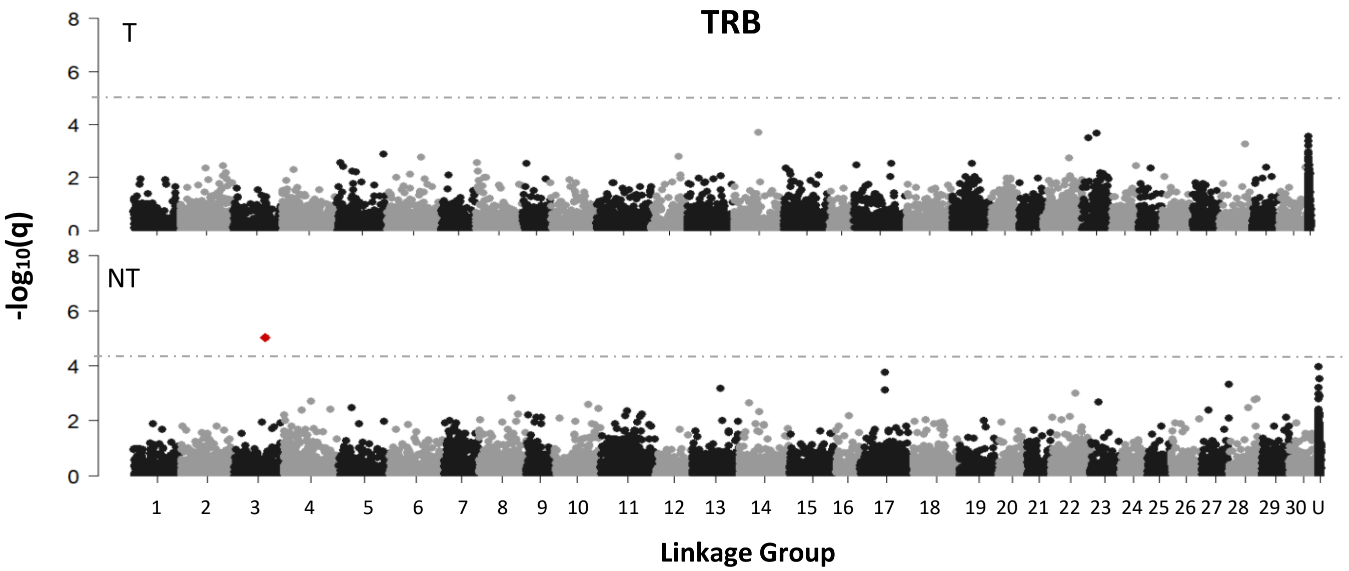
TRB
T
NT
-log10(q)
8
6
4
2
0
1 2 3 4 5 6 7 8 9 10 11 12 13 14 15 16 17 18 19 20 21 22 23 24 25 26 27 28 29 30 U
Linkage Group

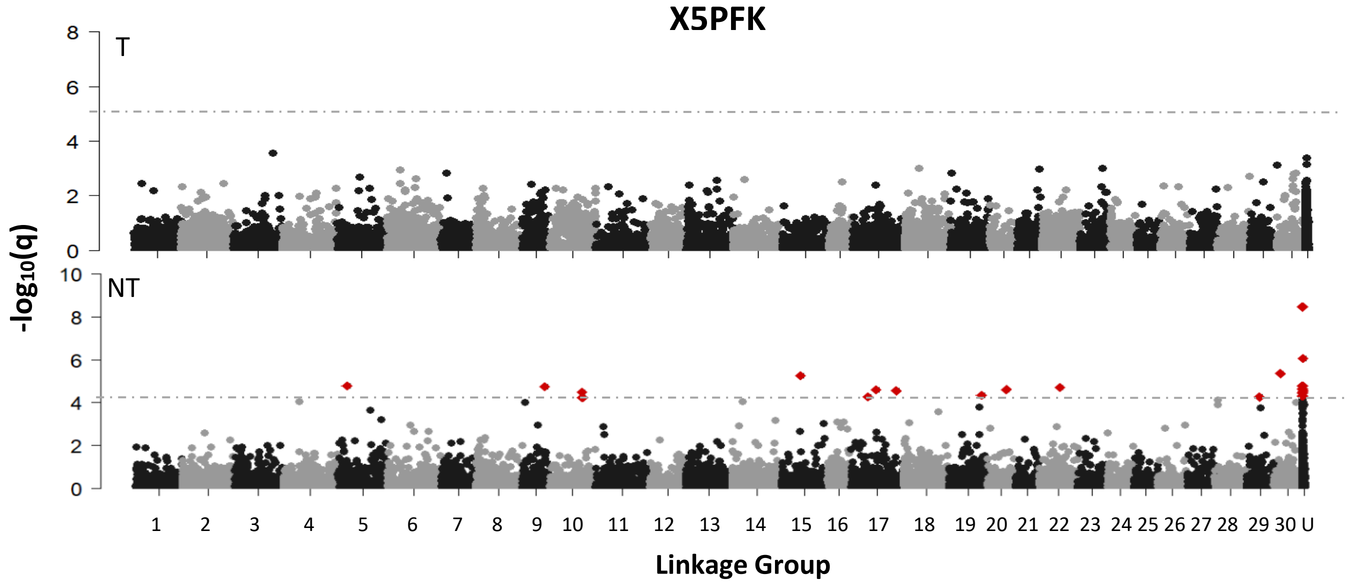
X5PFK
T
NT
-log10(q)
10
8
6
4
2
0
1 2 3 4 5 6 7 8 9 10 11 12 13 14 15 16 17 18 19 20 21 22 23 24 25 26 27 28 29 30 U
Linkage Group

Figure S6. Manhattan plots of SNP number and linkage group (1-30, and unassigned (U)) for weight, length and condition factor in transgenic (T) and non-transgenic (NT) fish. Significant SNPs (FDR=0.05) are indicated by red triangles; the dotted line indicates the significant q value.

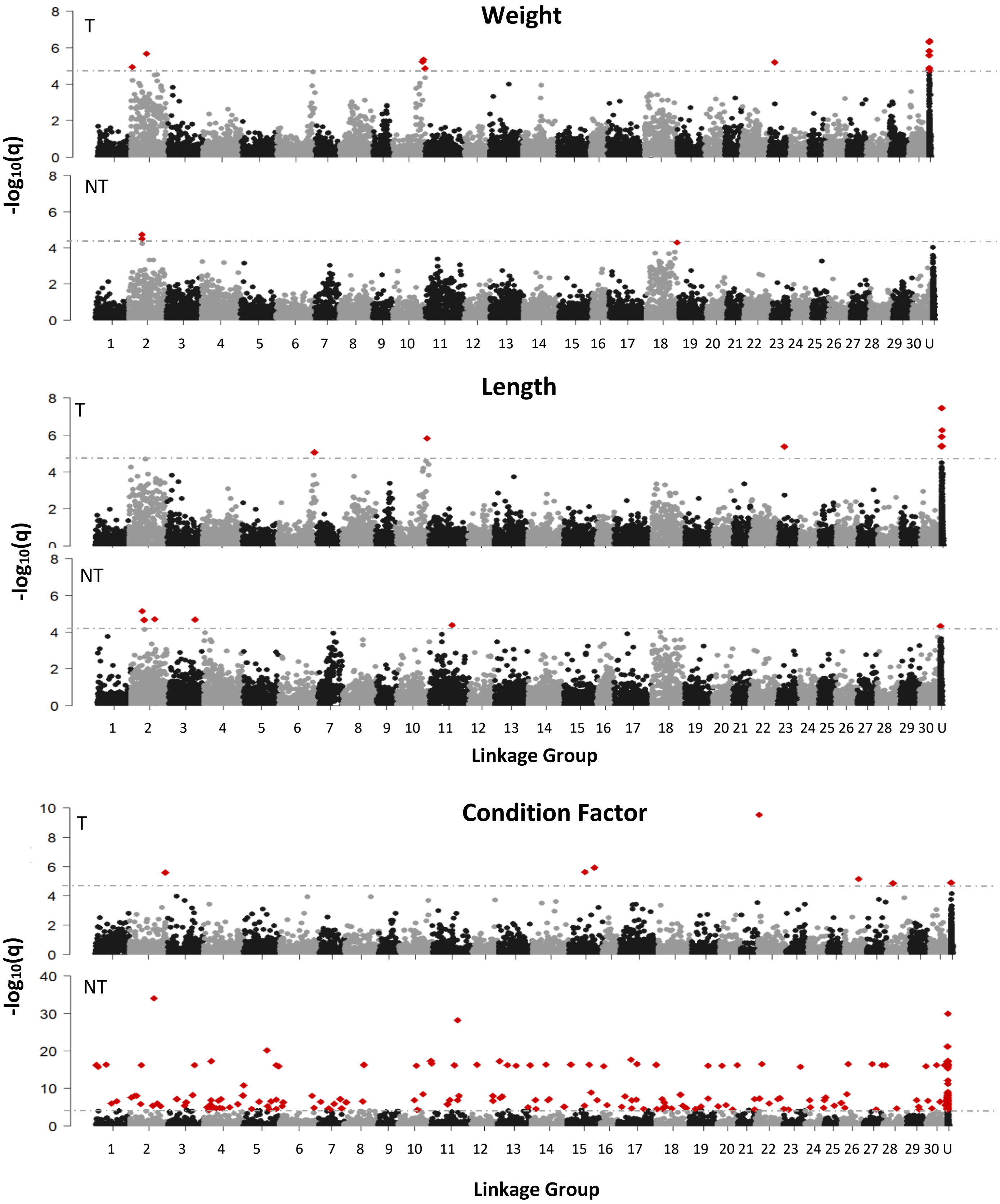

Supplement: Supplementary file 2 — Additional file 2: Figure S1. Gene Ontology (GO) Biological Process categories for the differentially expressed genes (DEGs) identified in comparisons between transgenotypes (transgenic fish, T, and non-transgenic fish, NT) for large and small fish. Figure S2. Box plots represent the median and 25% quantiles for relative gene expression for large and small transgenic (TLarge; TSmall) and nontransgenic (NT Large and NTSmall) fish. Groups with the different letters are significantly different (Tukey HSD, p < 0.05). Gene abbreviations are as for Table S2. Figure S3. Distribution of SNPs across Coho Salmon linkage groups. Figure S4. Histogram of lengths (cm) for transgenic (T) and non-transgenic (NT) fish at the time of sampling. A subset of these fish were used for further analysis as described in the text. Figure S5. Manhattan plots of SNP number and linkage group (1–30, and unassigned (U)) for expression traits in transgenic (T) and non-transgenic (NT) fish. Significant SNPs (FDR = 0.05) are indicated by red triangles; dotted line indicates significant q value. Name abbreviations are as for Table S2. Figure S6. Manhattan plots of SNP number and linkage group (1–30, and unassigned (U)) for weight, length and condition factor in transgenic (T) and non-transgenic (NT) fish. Significant SNPs (FDR = 0.05) are indicated by red triangles; the dotted line indicates the significant q value. [file 12864_2020_6586_MOESM2_ESM.zip › Supplemental Figures/Supplemental Figures.pdf]
